# Common health assets protocol: a mixed-methods, realist evaluation and economic appraisal of how community-led organisations (CLOs) impact on the health and well-being of people living in deprived areas

**DOI:** 10.1136/bmjopen-2022-069979

**Published:** 2023-03-15

**Authors:** Rachel Mairi Baker, Mohasin Ahmed, Marcello Bertotti, John Cassidy, Rejoice Chipuriro, Emma Clewett, Cam Donaldson, Andrew Elders, Lee Ann Fenge, Julie Fox, Karen Galway, Aideen Gildea, Aileen McGuinness, Jennifer McLean, Sarkis Manoukian, Helen Mason, Antony Morgan, Jill Mulholland, Liam O'Hare, Andrew Paterson, Sam Porter, Jack Rendall, Michael J Roy, Peter Seaman, Merron Simpson, Artur Steiner, Michael P Kelly

**Affiliations:** 1 Yunus Centre for Social Business and Health, Glasgow Caledonian University, Glasgow, UK; 2 Glasgow Centre for Population Health, Glasgow, UK; 3 Institute for Connected Communities, University of East London, London, UK; 4 Scottish Communities for Health and Wellbeing, Glasgow, UK; 5 Department of Social Sciences and Social Work, Bournemouth University, Poole, UK; 6 NMAHP Research Unit, Glasgow Caledonian University School of Health and Life Sciences, Glasgow, UK; 7 Annexe Communities Glasgow, Glasgow, UK; 8 School of Nursing and Midwifery, Queen's University Belfast, Belfast, UK; 9 Bogside and Brandywell Health Forum, Derry, UK; 10 Innovation Zones, School of Social Science, Education and Social Work, Queen's University Belfast, Belfast, UK; 11 Scottish Community Development Centre, Glasgow, UK; 12 The Health Creation Alliance, UK; 13 Public Health and Primary Care, University of Cambridge, Cambridge, UK

**Keywords:** PUBLIC HEALTH, SOCIAL MEDICINE, HEALTH ECONOMICS

## Abstract

**Introduction:**

This research investigates how community-led organisations’ (CLOs’) use of assets-based approaches improves health and well-being, and how that might be different in different contexts. Assets-based approaches involve ‘doing with’ rather than ‘doing to’ and bring people in communities together to achieve positive change using their own knowledge, skills and experience. Some studies have shown that such approaches can have a positive effect on health and well-being. However, research is limited, and we know little about which approaches lead to which outcomes and how different contexts might affect success.

**Methods and analysis:**

Using a realist approach, we will work with 15 CLOs based in disadvantaged communities in England, Scotland and Northern Ireland. A realist synthesis of review papers, and a policy analysis in different contexts, precedes qualitative interviews and workshops with stakeholders, to find out how CLOs’ programmes work and identify existing data. We will explore participants’ experiences through: a Q methodology study; participatory photography workshops; qualitative interviews and measure outcomes using a longitudinal survey, with 225 CLO participants, to assess impact for people who connect with the CLOs. An economic analysis will estimate costs and benefits to participants, for different contexts and mechanisms. A ‘Lived Experience Panel’ of people connected with our CLOs as participants or volunteers, will ensure the appropriateness of the research, interpretation and reporting of findings.

**Ethics and dissemination:**

This project, research tools and consent processes have been approved by the Glasgow Caledonian University School of Health and Life Sciences Ethics Committee, and affirmed by Ethics Committees at Bournemouth University, Queen’s University Belfast and the University of East London. Common Health Assets does not involve any National Health Service sites, staff or patients.

Findings will be presented through social media, project website, blogs, policy briefings, journal articles, conferences and visually in short digital stories, and photographic exhibitions.

**STRENGTHS AND LIMITATIONS OF THIS STUDY**

- Using a realist approach means that this study will build explicitly on theory and practice to generate understanding of what works, for whom, how and in what circumstances.
- A lived experience panel with input into design by community partners and stakeholders means that methods can be adjusted and improved in real time.
- Use of mixed methods will provide insights from different perspectives and test programme theories quantitatively as well as qualitatively.
- Our combination of realist and economic evaluation techniques will contribute towards knowledge in an area of ongoing methodological innovation.

## Introduction

This research focuses on place-based, community-owned organisations working in disadvantaged areas, which we label community-led organisations (CLOs). CLOs have a critical role in the delivery of health and social care, tackling health inequalities and underlying social determinants of health; for example, reducing loneliness and isolation, and increasing individual and community capacity for democratic participation.^1^ CLOs work in ways that have come to be known as an assets-based approaches, which involve doing with rather than doing to, and bring people in communities together to achieve positive change using their own knowledge, skills and experience. In the last decade, there has been an emergence of terminology around assets-based approaches in community development and health. The premise is that sustained positive health and social outcomes occur when people and communities have opportunities and facilities to manage their own futures.^2^

CLOs in the VCSE (voluntary, community and social enterprise) sector^3^ work in partnership with other organisations and networks, local government or health professionals. They are part of a developing field of collaborative public health initiatives that have received sustained policy attention^4–7^ but evidence of their effectiveness remains limited. A recent systematic review^8^ found that there is a variety of definitions of assets-based approaches, and that evidence in relation to health effects is scarce, largely presented in grey literature^9 10^ with a predominance of case studies. This is replicated by reviews of community development and health literature,^11 12^ which have observed that experimental approaches and controlled trials are often challenging when controlling ‘exposure’ is difficult and ‘interventions’ are complex.^13^ Despite this lack of robust evidence, previous research on the work of the VCSE sector (eg, the CommonHealth research programme^14–17^) provides a solid base for rigorous, theory-based evaluation to explore the contexts and mechanisms through which community-led approaches lead to improvements in health and well-being outcomes.

The Common Health Assets project will investigate the impact of community-led approaches on health and well-being in areas facing significant health and social inequalities, using a realist approach to determine ‘what works, for whom and in what circumstances’.^18^

The project addresses the following research objectives:

1. To develop, with stakeholders, initial realist programme theories, to explain ‘what works, for whom in what circumstances and how’ in relation to CLOs’ impact on health and well-being and health inequalities.
2. To test and refine programme theories by locating existing data and generating mixed method evidence to identify context–mechanism–outcome configurations (CMOCs).
3. To estimate the resource use and outcomes associated with different CMOCs in an economic appraisal.
4. To analyse CLO income streams and stakeholders’ views on sustainability and scalability.

## Methods and analysis

### Study design

The overarching methodology for this study is realist evaluation. Realist evaluation is a theory-driven form of evaluation which recognises that not every intervention will work for each person in the same way.^18^ Realist texts refer to the identification of CMOCs as programme theories, which can be tested and refined. Following Porter,^19^ we distinguish contextual mechanisms (CMs), which relate to factors within the social and organisational environment that influence people’s attitudes and behaviour, from programme mechanisms (PMs), which are the factors embedded in particular interventions designed to alter people’s attitudes and behaviour. We include agency (A), which is the capacity of actors to interpret, evaluate and respond to external social influences. The combination of contextual, programme and agential mechanisms produces outcomes (O). Outcomes of interest include both changes in behaviour over time, and effects on the flourishing or suffering of those involved.

**Figure 1:**
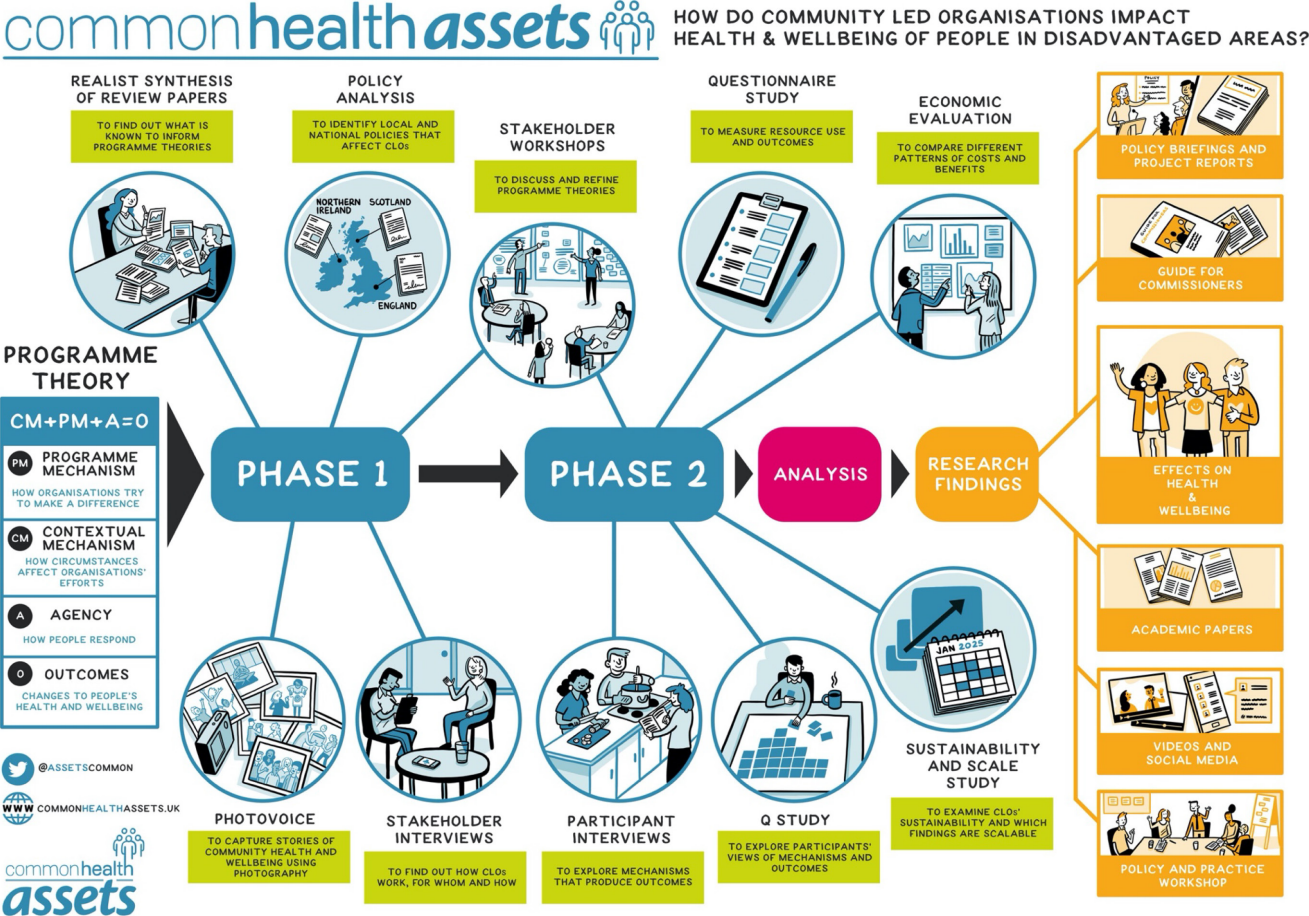
Study illustration: phase 1 and phase 2 methods and outputs.

This multimethod, realist study is designed in two phases as shown in figure 1—Study Illustration. In the first phase (employing realist synthesis, participatory photography workshops and qualitative methods) we will develop and refine programme theories describing CLOs’ impact on health and well-being, focusing on the CM and programme mechanisms that affect outcomes in different ways for different groups of people. In the second phase, we will test the programme theories by: using a longitudinal participant survey, participant interviews and Q-sort methods; examining the economic implications of findings in terms of the relationship between resources and outcomes of a range of programme theories; and exploration of sustainability and scalability of CLOs.

**Figure 2:**
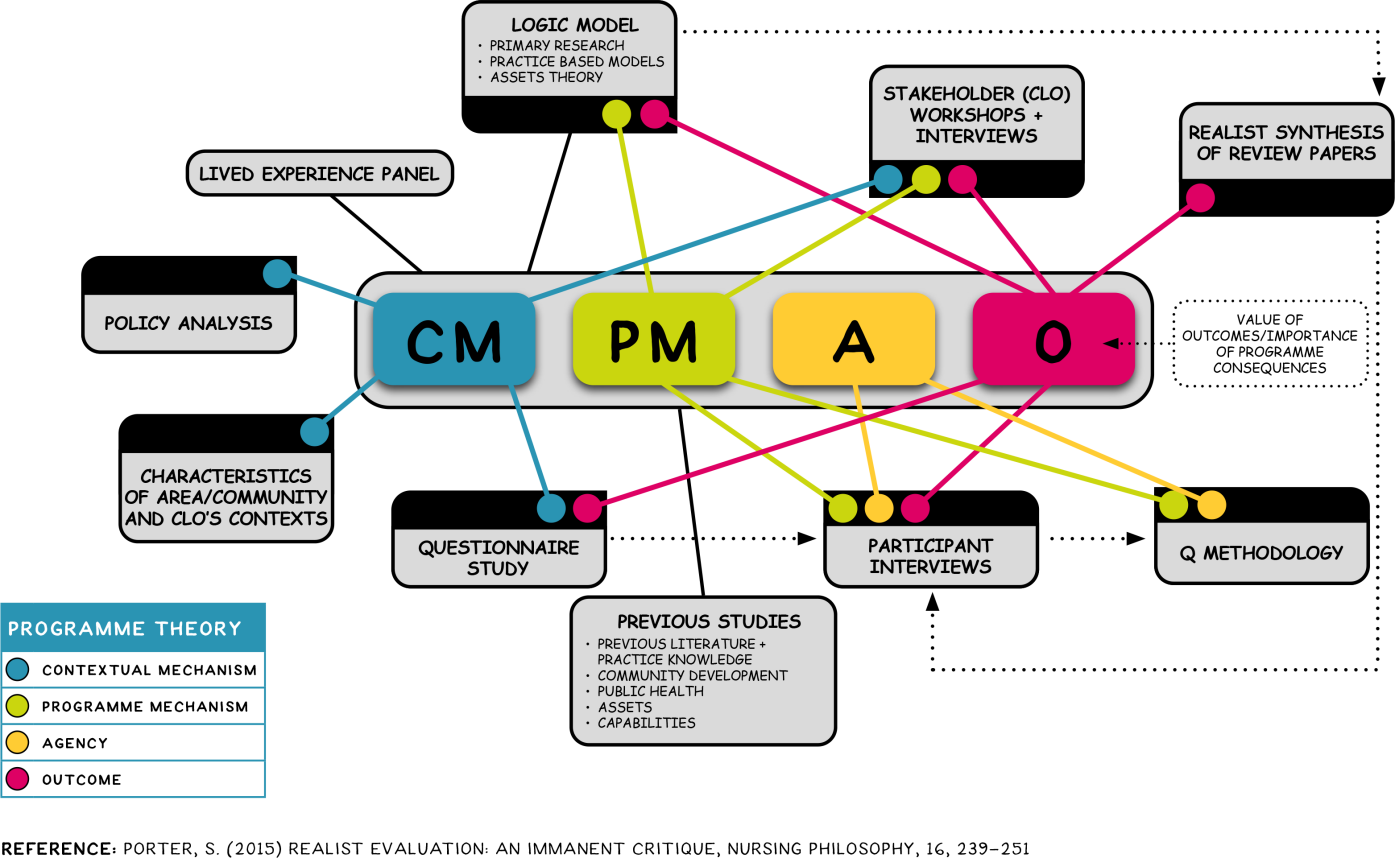
‘Plumbing diagram’ showing how data sources feed into the realist framework connecting context, mechanisms and outcomes.

Figure 2 is a ‘plumbing diagram’ showing the contribution of different sources of information and how the various study components and data sources contribute to our understanding of CM and PMs, actors’ responses and outcomes, and the evolving programme theory.

### Study setting and participants

The project will be conducted in Scotland, Northern Ireland and England. We will work with CLOs in communities with multiple disadvantages. This will be defined here as CLOs working with communities, and with people, in Index of Multiple Deprivation (IMD) deciles 1–3, that is, 10%–30% most deprived, based on the IMDs for each country.^20–22^ A researcher will be appointed in each area to work closely with CLOs. Approximately 15 CLOs will be recruited to the study. Although CLOs are similar in their focus on disadvantaged communities and in many of their approaches, we will select CLOs as research partners with the aim of achieving variation over a range of attributes including: geography and demography; CLO size and funding sources; characteristics of participant populations; premises or other physical assets; number of volunteers and professionals; range of activities available; relationships with local government, social and health services.

Recruitment of participants for all study components will be through the 15 CLOs involved in the research. For qualitative methods (see figures 1 and 2), including interviews, workshops, participatory photography and storytelling workshops, and Q sorting, sampling will be initially driven to achieve diversity through maximum variation, and subsequently more purposive—identifying key participants who might shine light on programme theories or offer different perspectives. Sampling for the survey will identify participants in CLO activities based on the inclusion and exclusion criteria outlined below and invite them to take part in the research by completing questionnaires at baseline and follow-up, as described under phase 2 below.

### Phase 1: developing and refining programme theories

**Figure 3:**
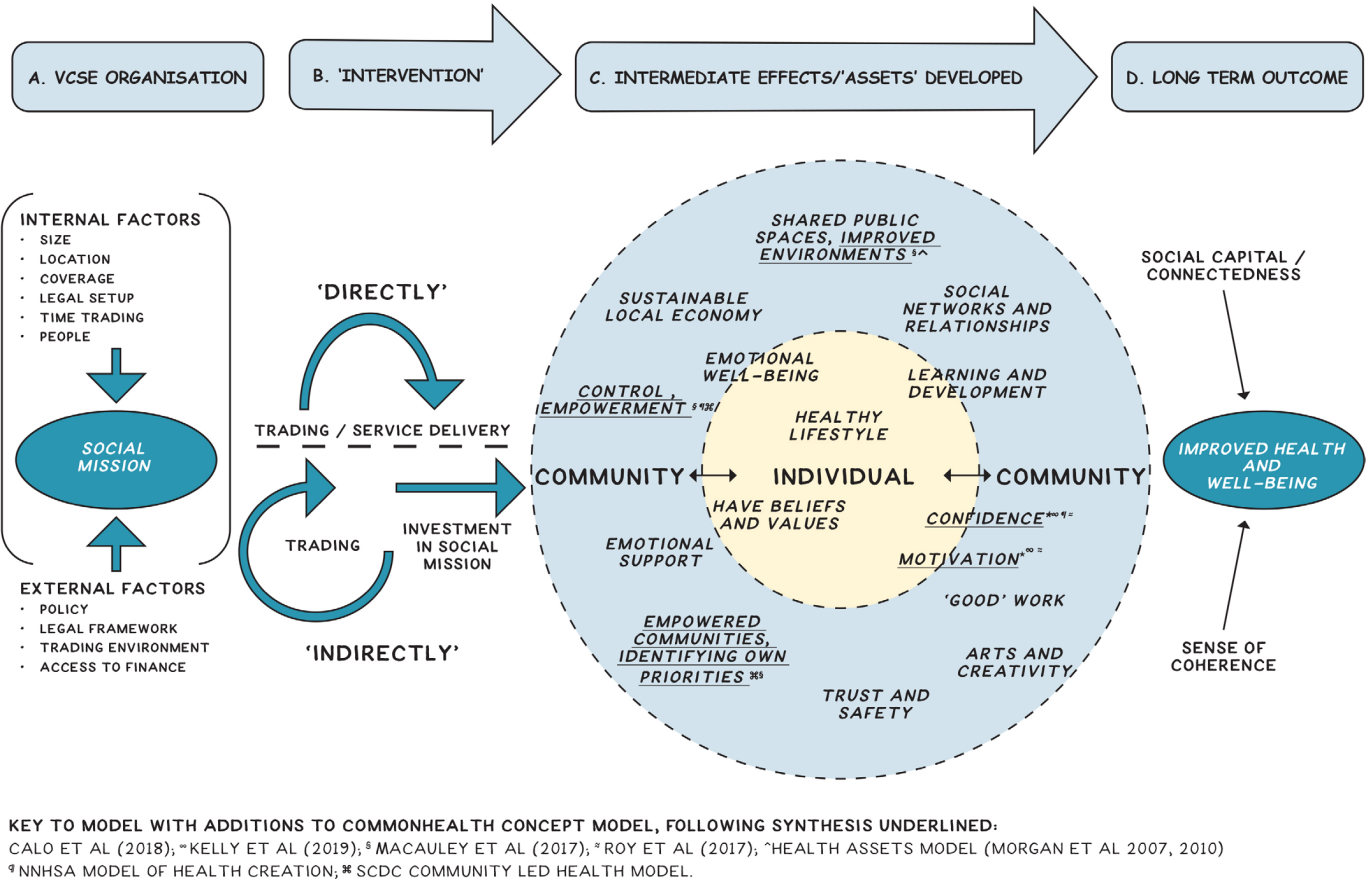
Conceptual model—a synthesis of research and practice-based models.

Our starting point is represented by a conceptual model that was developed based on a the ‘CommonHealth’ programme of research (http://www.commonhealth.uk/). This has been augmented with findings from qualitative research;^14 23^ assets theory^3 24 25^ and practice-based models of health creation and community development^10 26 27^ (figure 3 conceptual model). The model sets out the potential pathways of effect, from CLOs’ activities through to changes in health and well-being. It does not set out the ways in which impacts are realised or how CM or PMs might lead to different agents’ responses and outcomes. From this starting point, we will gather information from a number of sources to propose and refine programme theories and set out the CM and PMs likely to impact on actors and outcomes.

A realist synthesis of literature reviews (peer-reviewed and grey literature) will extract potential contextual factors, programme mechanisms and outcomes. Further details on the realist synthesis are provided in PROSPERO.^28^

Policy analysis will draw on policy documents identified as relevant in the contexts in which the CLOs are embedded. Interviews and focus groups with CLO staff will explore how the policy and funding contexts differ in each setting, and how this potentially impacts on CLOs and on outcomes. A purposive sample will be selected from CLO staff at different level of the organisation and other key informants with influence on the work of CLOs. The analysis will focus on actions and activities that are policy driven or policy contingent (including barriers to action and unintended consequences of policies) to reveal potential (outer) CMs that might inhibit or enhance outcomes. The nature of relationships between the local public sector and CLOs will be explored, for instance, differences between policy rhetoric and reality; the availability and stability of funding; whether different attitudes exist towards CLOs to address local social vulnerabilities in each of the different contexts; and whether such differences matter.

Participatory photography workshops with CLO participants will focus on CMs and how communities and CLOs create the conditions or barriers to health and well-being^29^ (see www.photovoice.org). These workshops will elicit community participants’ views of what creates or prevents good health and well-being in their communities. These workshops provide community members with the opportunity to learn about photography and create images that represent, within their communities, those things that contribute to or detract from their well-being, and tell a story about what each image means.

Stakeholder interviews and workshops will build on the realist review and policy analysis, to explore the design of programmes in CLOs, and what works, for whom and how, to produce health and well-being outcomes. In phase 1, we will engage with 40–60 stakeholders in interviews and workshops.

Qualitative data will be coded in terms of Contextual Mechanisms, Programme Mechanisms, Agency and Outcomes. Initial programme theories will be refined by referring to codes that index data in this way, always focusing on what works, for whom, in what contexts. Importantly, there will be unintended outcomes, positive and negative, and data will be searched for confirmatory as well as divergent patterns. Qualitative data will be transcribed verbatim and coded using NVivo^30^ initially using broad ‘bucket coding’ to identify themes in the data^31^ and thereafter in relation to programme theories.

As programme theories are refined, and with a view to exploring patterns in the qualitative data across cases and sites, we will employ qualitative comparative analysis (QCA) to enable decisive cross-case patterns to be identified, which is the usual domain of quantitative analysis. QCA respects the heterogeneity of the contexts and different causally relevant conditions by comparing cases as configurations. This method will help to systematise our qualitative analysis across four research sites, working with four researchers, 15 CLOs multiple participants and stakeholders. We will produce statements of the combination of CMs, PMs and agents’ responses that lead to improvements in health and well-being outcomes. Where there are differences in outcomes, we will seek to identify the variations in configurations of mechanisms that lead to differing results.

This early work, together with input from our lived experience panel (LE), will be used to develop and refine programme theories. These will then be presented to stakeholders in interviews and finally in workshops with a view to further refining. These initial programme theories will be tested in phase 2.

### Phase 2: testing programme theories, economic analysis, sustainability and scale

#### Questionnaire study

A longitudinal questionnaire will generate data about new participants in CLOs focusing on resource use and outcomes at baseline, 1, 6 and 12 months. Questionnaires will be administered in person, by telephone or otherwise remotely (using MS Teams and REDCap software) to maximise response rates. For consistency and comparability, follow-up questionnaires will adopt the same mode of administration as baseline, as far possible. Eligibility criteria for the questionnaire study are shown in table 1.

**Table 1:** Eligibility criteria for questionnaire study

Inclusion criteria	Exclusion criteria
Aged over 18 years	Ongoing participation in multiple CLOs
Community participant associated with participating CLOs	One-time participation only
Participant in an activity that involves several contacts over time (CLOs might provide one-time advice services eg, but, for comparability, we will focus on a period of consistent participation).	Not within the community of the CLO.

CLO, community-led organisation.

Eligible participants who express interest in taking part in the study via the CLO staff will be contacted by researchers to provide study information, complete consent procedures and administer a baseline questionnaire comprised of sociodemographic questions, resource use questions and a number of standardised outcome measures.

The primary outcome measure for the questionnaire study is the ICECAP-A (ICEpop CAPability measure for adults).^32^ The ICECAP-A has been selected for three important reasons. First, the dimensions of the ICECAP-A map well onto the intermediate outcomes identified in previous empirical research and in practice-based models synthesised in our conceptual model (see figure 3) and so we set out to measure things that have been shown to be important to beneficiaries, practitioners and according to theory. Second it is validated and is supported for use in economic evaluation by NICE^33^ and thirdly preference-based population tariffs are available.^34^ Our secondary outcome measures are health status using EQ-5D 5L,^35^ Warwick Edinburgh Mental Well-being Scale (WEMWBS),^36^ and Social Connectedness.^37^

There is no estimate of minimal important difference available from existing datasets of ICECAP-A, so the study will be designed to detect a commonly used standardised effect size of 0.25 at 6 months. This represents a small to medium effect size. A sample size of 252 will be required to provide 80% power at 5% significance (with two-sided alpha) to detect a difference of 0.25 SDs. We will recruit 360 to achieve an effective sample of 252 completed questionnaires at 6 months, which allows for credible retention of 70%. We anticipate 60%–65% retention at 12 months (n=225).

A statistical analysis plan will be agreed before database lock. Descriptive statistics will be used to explore and analyse the data for associations. Random effects regressions with clustered standard errors will be employed to account for the longitudinal nature of our data and for the fact individuals are clustered in CLOs. Multilevel modelling that explicitly acknowledges the hierarchical clustering of the data will be employed.^38 39^ These models will estimate the impact of the CLOs on the outcome measures over time after controlling for individual and CLO characteristics. The analyses will be run for our primary and secondary outcomes separately.

#### Q methodology study

The aim of all Q studies is to identify patterns of shared perspectives.^40–42^ In the context of realist methods this sits well with the goal of uncovering regularities in data.^43^ We will make use of this approach to unpack the mechanisms at work in different contexts.^44^ The Q set of statements will comprise of candidate mechanisms drawn from theory, interview and workshop data and hypotheses that emerge from preliminary analysis of context-outcome patterns in the questionnaire study.

Up to 60 participants, selected from the questionnaire sample to cover different contexts and outcomes, will complete Q sorts to identify which mechanisms they consider help to explain why outcomes arise (or do not) in context. Different mechanisms might relate to the same outcomes in different contexts and we will gather descriptive information about Q participants’ contexts. Factor analysis of Q sort data based on correlations between individuals’ Q sorts will reveal shared patterns of context and mechanisms for interpretation.

#### Economic analysis

The economic evaluation will take the form of a cost–consequence analysis (CCA). A CCA presents costs and outcomes in a disaggregated form, which is appropriate given the multisectoral context in which the CLOs operate and the range of outcomes of importance.^45^ We will seek to quantify resource implications arising from both CM and PM, and how these relate to agents’ responses (A) and outcomes (O) measured.

The first stage of the economic analysis will be to map out the resource inputs required to deliver the activities provided by the CLOs. This will be done in collaboration with the CLOs and will be assessed and updated throughout the project. All resource inputs will be totalled and turned into a cost per participant. Second, as part of the questionnaire study, participants will self-report use of other healthcare, social and community-based services in the month prior to joining the CLO and then at 1, 6 and 12 months follow-up. This will allow us to assess changes in resource use within the study participant group in the form of a before-and-after design. Units of each item will be recorded and presented along with unit costs deriving from local publicly available sources.^46^

The resource use will be presented alongside the estimates of health and well-being outcomes from the primary and secondary outcome measures and with qualitative data from the interviews conducted in both phases 1 and 2 of the study in the CCA.

#### Sustainability and scalability

Building on information deriving from our economic analysis and relevant financial documents (eg, contracts and agreements) provided by CLOs managers, our assessment of financial sustainability and scalability of CLOs will be informed by interviews with CLO stakeholders (eg, managers, funders). Combining economic evaluation with qualitative interview data will help to better understand processes associated with developing sustainable CLOs. The interviewees will be asked about best practices and key challenges involved in establishing, operating and growing sustainable CLOs, and what it means to be ‘sustainable’ in their specific community contexts. Previous research^47^ suggests that sustainability and scalability in a community context is rarely straightforward and CLOs frequently face difficulties in maintaining and sustaining their services and activities.

Drawing on a sample of diverse stakeholders from a range of CLOs (ie, variation across rural/urban, organisational size and maturity, range of activities) will help us to explore how different contexts can promote or inhibit particular mechanisms; intended and unintended outputs and outcomes; income streams and views about sustainability.

### Patient and public involvement

Public involvement in the preparation and design of the project as presented to funders was achieved through coinvestigators and collaborators in the community sector who work in and with CLOs and are coauthors on this paper.

Patient and public involvement and engagement (PPIE) has been integrated throughout the project by establishing a panel of individuals with experience of living in our CLO communities. The ‘LE panel’ will ensure that the project is informed and guided by ongoing community expertise, perspective and voice and that findings are relevant and meaningful to community organisations.

The LE panel will comprise representatives of the CLOs, identified and recruited with the support of our community partners. The LE panel will meet at key stages of the research process to guide and inform the research questions, methods, data analysis and interpretation of results—with space for ongoing involvement and communication between Panel meetings. The hands-on nature of these participatory methods and the support of the research team will allow participants to actively engage with the research, placing more value on their expertise. Representatives of the LE Panel will also be members of the study steering committee, complementing the perspectives of other key stakeholders.

Training opportunities will be provided allowing participants to build their skills and knowledge around community-based research, promoting two-way learning exchange between the researchers and Panel participants, which will elevate community voices and strengthen researcher–participant relations. Continuous evaluation will be fostered to improve facilitation and promote learning throughout the life and work of the Panel.

### Ethics and dissemination

This project has been approved by the Glasgow Caledonian University School of Health and Life Sciences Ethics Committee on 15 July 2021 (ref HLS/NCH/20/034) and subsequently affirmed by Ethics Committees at Bournemouth University, Queens University Belfast (MHLS 21_94) and University of East London (ETH2021-0226). Common Health Assets does not involve any National Health Service (NHS) sites, staff or patients. IRAS and NHS REC are not required. The project is funded for 3 years beginning on 1 September 2021.

This project will advance the research field, at the nexus of public health research and community development/social enterprise research and methodologically in terms of economics and quantitative approaches in realist evaluation and in the use of Q methodology to explore mechanisms. Our PPIE strategy including lived LE, stakeholder involvement in the research team and Study Steering Committee, and policy briefing will ensure that the research involves users of evidence throughout to enhance the relevance and application to policy and practice. CLOs will be able to use research findings as part of their impact evidence base when applying for public funding or in refining their activities where appropriate. We will disseminate our findings using a range of media, and pitched to suit the needs of our three main constituencies of interest; namely: (1) practitioners and activists; (2) policy makers and commissioners; and (3) academics.

The project outputs will comprise peer-reviewed papers in academic journals, blogs, short videos, photography exhibition, policy briefing papers, website (www.commonhealthassets.uk) and a commissioning guide. We will hold workshops to share findings and develop recommendations with practitioners and policy actors. Each CLO will receive a report summarising the study findings and their own local findings where possible.

## Supplementary Material

Reviewer comments

Author's manuscript
