# Supplementary material for: Common health assets protocol: a mixed-methods, realist evaluation and economic appraisal of how community-led organisations (CLOs) impact on the health and well-being of people living in deprived areas
Source: BMJ Open. 2023 Mar 15;13(3):e069979. doi: 10.1136/bmjopen-2022-069979 (PMC10030928; doi:10.1136/bmjopen-2022-069979)
Supplement: Author's manuscript [file bmjopen-2022-069979.draft_revisions.pdf]

BMJ Open is committed to open peer review. As part of this commitment we make the peer review history of every article we publish publicly available.

When an article is published we post the peer reviewers' comments and the authors' responses online. We also post the versions of the paper that were used during peer review. These are the versions that the peer review comments apply to.

The versions of the paper that follow are the versions that were submitted during the peer review process. They are not the versions of record or the final published versions. They should not be cited or distributed as the published version of this manuscript.

BMJ Open is an open access journal and the full, final, typeset and author-corrected version of record of the manuscript is available on our site with no access controls, subscription charges or pay-per-view fees (http://bmjopen.bmj.com).

If you have any questions on BMJ Open's open peer review process please email info.bmjopen@bmj.com

BMJ Open

# Common Health Assets protocol: a mixed-methods, realist evaluation and economic appraisal of how community-led organisations (CLOs) impact on the health and wellbeing of people living in deprived areas

| | |
|---|---|
| Journal: | *BMJ Open* |
| Manuscript ID | bmjopen-2022-069979 |
| Article Type: | Protocol |
| Date Submitted by the Author: | 10-Nov-2022 |
| Complete List of Authors: | Baker, Rachel; Glasgow Caledonian University, Yunus Centre for Social Business and Health<br>Ahmed, Mohasin; Glasgow Centre for Population Health<br>Bertotti, Marcello; University of East London, Institute for Connected Communities<br>Cassidy, John; Scottish Communities for Health and Wellbeing<br>Chipuriro, Rejoice; Bournemouth University<br>Clewett, Emma; University of East London<br>Donaldson, Cam; Glasgow Caledonian University, Yunus Centre for Social Business and Health<br>Elders, A; Glasgow Caledonian University School of Health and Life Sciences, NMAHP Research Unit ; Glasgow Caledonian University<br>Fenge, Lee Ann; Bournemouth University<br>Fox, Julie; Annexe Communities Glasgow<br>Galway, K.; Queen's University Belfast<br>Gildea, Aideen; Queen's University Belfast<br>McGuiness, Aileen; Bogside and Brandywell Health Forum<br>McLean, Jennifer; Glasgow Centre for Population Health<br>Manoukian, Sarkis; Glasgow Caledonian University, Yunus Centre for Social Business and Health<br>Mason, Helen; Glasgow Caledonian University, Yunus Centre for Social Business and Health<br>Morgan, A; Glasgow Caledonian University London, Yunus Centre for Social Business and Health<br>Mulholland, Jill; Queen's University Belfast<br>O'Hare, Liam; Queens University of Belfast<br>Paterson, Andrew; Scottish Community Development Centre<br>Porter, Sam; Bournemouth University, Social Sciences and Social Work<br>Rendall, Jack; Glasgow Caledonian University, Yunus Centre for Social Business and Health<br>Roy, Michael; Glasgow Caledonian University,<br>Seaman, Peter; Glasgow Centre for Population Health<br>Simpson, Merron; The Health Creation Alliance<br>Steiner, Artur; Glasgow Caledonian University, Yunus Centre for Social Business and Health<br>Kelly, Michael; University of Cambridge, Public Health and Primary Care |
| Keywords: | PUBLIC HEALTH, SOCIAL MEDICINE, HEALTH ECONOMICS |

SCHOLARONE™
Manuscripts

## Title

Common Health Assets protocol: a mixed-methods, realist evaluation and economic appraisal of how community-led organisations (CLOs) impact on the health and wellbeing of people living in deprived areas

## Corresponding Author

Professor Rachel Baker PhD
Yunus Centre for Social Business and Health
Glasgow Caledonian University
70 Cowcaddens Road
Glasgow G4 0BA
Rachel.Baker@gcu.ac.uk
Tel: 0141 331 8246
Fax: n/a

## Co Authors

Miss Mohasin Ahmed, Glasgow Centre for Population Health, Mohasin.Ahmed@glasgow.ac.uk (Glasgow, Scotland)
Dr Marcello Bertotti PhD, Institute for Connected Communities, University of East London, m.bertotti@uel.ac.uk (London, England)
Dr John Cassidy, Scottish Communities for Health and Wellbeing, john.kcassidy@ntlworld.com (Glasgow, Scotland)
Dr Rejoice Chipuriro PhD, Institute for Connected Communities, Bournemouth University, rchipuriro@bournemouth.ac.uk (Bournemouth, England)
Miss Emma Clewett, University of East London, eclewett@uel.ac.uk (London, England)
Professor Cam Donaldson PhD FRSE, Yunus Centre for Social Business and Health, GCU, Cam.Donaldson@gcu.ac.uk (Glasgow, Scotland)
Dr Andrew Elders, NMAHP Research Unit, GCU Andrew.Elders@gcu.ac.uk (Glasgow, Scotland)
Professor Lee Ann Fenge, Bournemouth University, lfenge@bournemouth.ac.uk (Bournemouth, England)
Ms Julie Fox, Annexe Communities Glasgow, Julie.fox@annexecommunities.org.uk (Glasgow, Scotland)
Dr Karen Galway, Queen's University Belfast, k.galway@qub.ac.uk (Belfast, Northern Ireland)
Dr Aideen Gildea, Queen's University Belfast, a.gildea@qub.ac.uk (Belfast, Northern Ireland)
Miss Aileen McGuinness, Bogside and Brandywell Health Forum Derry, aileen@bbhealthforum.org (Derry, Northern Ireland)
Dr Jennifer McLean PhD, Glasgow Centre for Population Health, jennifer.mclean@glasgow.ac.uk (Glasgow, Scotland)
Dr Sarkis Manoukian PhD, Yunus Centre for Social Business and Health, GCU Sarkis.Manoukian@gcu.ac.uk (Glasgow, Scotland)
Professor Helen Mason PhD, Yunus Centre for Social Business and Health, GCU Helen.Mason@gcu.ac.uk (Glasgow, Scotland)
Professor Antony Morgan PhD, Yunus Centre, GCU London, Antony.Morgan@gcu.ac.uk (London, England)
Ms Jill Mullholland, Queen's University Belfast, jmulholland18@qub.ac.uk (Belfast, Northern Ireland)

For peer review only

Dr Liam O'Hare, School of Social Sciences, Education and Social Work, Queen's University Belfast, L.OHare@qub.ac.uk (Belfast, Northern Ireland)
Dr Andrew Paterson, Scottish Community Development Centre, andrew.paterson@scdc.org.uk (Glasgow, Scotland)
Professor Sam Porter, Bournemouth University, porters@bournemouth.ac.uk (Bournemouth, England)
Dr Jack Rendall PhD, Yunus Centre for Social Business and Health, GCU Jack.Rendall@gcu.ac.uk (Glasgow, Scotland)
Professor Michael J Roy PhD, Yunus Centre for Social Business and Health, GCU Michael.Roy@gcu.ac.uk (Glasgow, Scotland)
Dr Peter Seaman PhD, Glasgow Centre for Population Health, peter.seaman@glasgow.ac.uk (Glasgow, Scotland)
Ms Merron Simpson, Health Creation Alliance, merron@thehealthcreationalliance.org (Birmingham, England)
Professor Artur Steiner PhD, Yunus Centre for Social Business and Health, GCU Artur.Steiner@gcu.ac.uk (Glasgow, Scotland)
Professor Michael P Kelly, University of Cambridge, mk744@medschl.cam.ac.uk (Edinburgh, Scotland)

## Abstract

### Introduction

This research investigates how community-led organisations' (CLOs') use of *assets-based approaches* improves health and wellbeing, and how that might be different in different contexts. Assets based approaches involve 'doing with' rather than 'doing to' and bring people in communities together to achieve positive change using their own knowledge, skills, and experience. Some studies have shown that such approaches can have a positive effect on health and wellbeing. However, research is limited, and we know little about which approaches lead to which outcomes, and how different contexts might affect success.

### Methods and analysis

Using a realist approach, we will work with 15 CLOs based in disadvantaged communities in England, Scotland, and Northern Ireland. A realist synthesis of review papers, and a policy analysis in different contexts, precedes qualitative interviews and workshops with stakeholders, to find out how CLOs' programmes work, and identify existing data. We will explore participants' experiences through: a Q methodology study; participatory photography workshops; qualitative interviews; and measure outcomes using a longitudinal survey, with 225 CLO participants, to assess impact for people who connect with the CLOs. An economic analysis will estimate costs and benefits to participants, for different contexts and mechanisms. A 'Lived Experience Panel' of people connected with our CLOs as participants or volunteers, will ensure the appropriateness of the research, interpretation and reporting of findings.

### Ethics and dissemination

This project, research tools and consent processes have been approved by the Glasgow Caledonian University School of Health and Life Sciences Ethics Committee, and affirmed by

For peer review only

Ethics Committees at Bournemouth University, Queen's University Belfast, and the University of East London. Common Health Assets does not involve any NHS sites, staff, or patients. Findings will be presented through social media, project website, blogs, policy briefings, journal articles, conferences, and visually in short digital stories, and photographic exhibitions.

## Abstract word count

341


## Keywords

Community development, community resources, evaluation study, quality of life, social determinants of health


## Word count

3745

## Article Summary

## Strengths and Limitations of this study

- This realist study design builds on theory and practice to generate understanding of what works, for whom, how and in what circumstances.
- Our sample of different types of CLOs in Scotland, England and Northern Ireland compares outcomes across a range of UK contexts.
- Our lived experience panel, with input into design by partners and stakeholders throughout the study, aims to improve research techniques, understanding and interpretation of findings.
- Mixed methods provide insights from different perspectives and test programme theories quantitatively as well as qualitatively.
- Our combination of realist and economic evaluation techniques will contribute towards knowledge in an area of ongoing methodological innovation.

## Introduction

This research focusses on place-based, community-owned organisations working in disadvantaged areas, which we label Community-Led Organisations (CLOs). CLOs have a critical role in the delivery of health and social care, tackling health inequalities and underlying social determinants of health; for example reducing loneliness and isolation, and increasing individual and community capacity for democratic participation[1]. CLOs work in ways that have come to be known as an assets-based approaches, which involve *doing with* rather than *doing to*, and bring people in communities together to achieve positive change using their own knowledge, skills, and experience. In the last decade there has been an emergence of terminology around assets-based approaches in community development and health. The

For peer review only

premise is that sustained positive health and social outcomes occur when people and communities have opportunities and facilities to manage their own futures[2].

CLOs in the VCSE (voluntary, community, and social enterprise) sector[3] work in partnership with other organisations and networks, local government or health professionals. They are part of a developing field of collaborative public health initiatives that have received sustained policy attention[4–7] but evidence of their effectiveness remains limited. A recent systematic review[8] found that there is a variety of definitions of assets-based approaches, and that evidence in relation to health effects is scarce, largely presented in grey literature[9,10] with a predominance of case studies. This is replicated by reviews of community development and health literature,[11,12] which have observed that experimental approaches and controlled trials are often challenging when controlling 'exposure' is difficult and 'interventions' are complex[13]. Despite this lack of robust evidence, previous research on the work of the voluntary, community and social enterprise (VCSE) sector (e.g. the CommonHealth research programme[14–17]) provides a solid base for rigorous, theory-based evaluation to explore the contexts and mechanisms through which community-led approaches lead to improvements in health and wellbeing outcomes.

The Common Health Assets project will investigate the impact of community-led approaches on health and wellbeing in areas facing significant health and social inequalities, using a realist approach to determine 'what works, for whom, and in what circumstances'[18].

The project addresses the following research objectives:

1. To develop, with stakeholders, initial realist programme theories, to explain 'what works, for whom in what circumstances and how' in relation to CLOs' impact on health and wellbeing and health inequalities
2. To test and refine programme theories by locating existing data and generating mixed method evidence to identify context-mechanism-outcome configurations (CMOCs)
3. To estimate the resource use and outcomes associated with different CMOCs in an economic appraisal
4. To analyse CLO income streams and stakeholders' views on sustainability and scalability

## Methods and Analysis

### Study Design

The overarching methodology for this study is realist evaluation. Realist evaluation is a theory driven form of evaluation which recognises that not every intervention will work for each person in the same way[18]. Realist texts refer to the identification of context-mechanism-outcome configurations (CMOCs) as programme theories which can be tested and refined. Following Porter[19], we distinguish contextual mechanisms (CMs), which relate to factors within the social and organisational environment that influence people's attitudes and behaviour, from programme mechanisms (PM), which are the factors embedded in particular interventions designed to alter people's attitudes and behaviour. We include agency (A),

For peer review only

which is the capacity of actors to interpret, evaluate and respond to external social influences. The combination of contextual, programme and agential mechanisms produces outcomes (O). Outcomes of interest include both changes in behaviour over time, and effects on the flourishing or suffering of those involved.

This multi-method, realist study is designed in two phases as shown in Figure 1 – Study Illustration. In the first phase (employing realist synthesis, participatory photography workshops, and qualitative methods) we will develop and refine programme theories describing CLOs' impact on health and wellbeing, focussing on the contextual and programme mechanisms that affect outcomes in different ways for different groups of people. In the second phase we will test the programme theories by: using a longitudinal participant survey, participant interviews and Q-sort methods; examining the economic implications of findings in terms of the relationship between resources and outcomes of a range of programme theories; and exploration of sustainability and scalability of CLOs.

****Insert figure one around here****

Figure 2 is a 'Plumbing Diagram' showing the contribution of different sources of information and how the various study components and data sources contribute to our understanding of contextual and programme mechanisms, actors' responses and outcomes, and the evolving programme theory.

****Insert figure two around here****

## Study Setting and participants

The project will be conducted in Scotland, Northern Ireland, and England. We will work with CLOs in communities with multiple disadvantages. This will be defined here as CLOs working in communities, and with, people in Index of Multiple Deprivation (IMD) deciles 1-3: that is, 10% to 30% most deprived, based on the IMDs for each country[20–22]. A researcher will be appointed in each area to work closely with CLOs. Approximately 15 CLOs will be recruited to the study. Although CLOs are similar in their focus on disadvantaged communities and in many of their approaches, we will select CLOs as research partners with the aim of achieving variation over range of attributes including: geography and demography; CLO size and funding sources; characteristics of participant populations; premises or other physical assets; number of volunteers and professionals; range of activities available; relationships with local government, social and health services.

Recruitment of participants for all study components will be through the 15 CLOs involved in the research. For qualitative methods (see Figures 1 and 2), including interviews, workshops, participatory photography and storytelling workshops, and Q sorting, sampling will be initially driven to achieve diversity through maximum variation, and subsequently more purposive – identifying key participants who might shine light on programme theories or offer different perspectives. Sampling for the survey will identify participants in CLO activities based on the inclusion and exclusion criteria outlined below and invite them to take part in the research by completing questionnaires at baseline and follow up, as described under phase 2 below.

For peer review only

## Phase 1: developing and refining programme theories

Our starting point is represented by a conceptual model that was developed based on a the 'CommonHealth' programme or research (http://www.commonhealth.uk/). This has been augmented with findings from qualitative research[14,23]; assets theory [3,24,25]; and practice-based models of health creation and community development[10,26,27] (Figure 3 Conceptual Model). The logic model sets out the potential pathways of effect, from CLOs' activities through to changes in health and wellbeing. It does *not* set out the ways in which impacts are realised or how contextual or programme mechanisms might lead to different agents' responses and outcomes. From this starting point we will gather information from a number of sources to propose and refine programme theories and set out the contextual and programme mechanisms likely to impact on actors and outcomes.

****Insert figure three around here****

A **realist synthesis of literature reviews** (peer reviewed and grey literature) will extract potential contextual factors, programme mechanisms and outcomes. Further details on the realist synthesis are provided in PROSPERO[28].

**Policy analysis** will draw on policy documents identified as relevant in the contexts in which the CLOs are embedded. Interviews and focus groups with CLO staff will explore how the policy and funding contexts differ in each setting, and how this potentially impacts on CLOs and on outcomes. A purposive sample will be selected from CLO staff at different level of the organisation and other key informants with influence on the work of CLOs. The analysis will focus on actions and activities that are policy-driven or -contingent (including barriers to action and unintended consequences of policies) to reveal potential (outer) contextual mechanisms that might inhibit or enhance outcomes. The nature of relationships between the local public sector and CLOs will be explored, for instance: differences between policy rhetoric and reality; the availability and stability of funding; whether different attitudes exist towards CLOs to address local social vulnerabilities in each of the different contexts; and whether such differences matter.

**Participatory photography workshops** with CLO participants will focus on contextual mechanisms and how communities and CLOs create the conditions or barriers to health and wellbeing[29] (See www.photovoice.org) . These workshops will elicit community participants' views of what creates or prevents good health and wellbeing in their communities. These workshops provide community members with the opportunity to learn about photography and create images that represent, within their communities, those things that contribute to or detract from their wellbeing, and tell a story about what each image means.

**Stakeholder interviews and workshops** will build on the realist review and policy analysis, to explore the design of programmes in CLOs, and what works, for whom and how, to produce health and wellbeing outcomes. In Phase 1 we will engage with 40-60 stakeholders in interviews and workshops.

Qualitative data will be coded in terms of Contextual Mechanisms, Programme Mechanisms, Agency and Outcomes. Initial programme theories will be refined by referring to codes that

For peer review only

index data in this way, always focusing on what works, for whom, in what contexts. Importantly there will be unintended outcomes, positive and negative, and data will be searched for confirmatory as well as divergent patterns. Qualitative data will be transcribed verbatim and coded using NVivo[30] initially using broad 'bucket coding' to identify themes in the data[31] and thereafter in relation to programme theories.

As programme theories are refined, and with a view to exploring patterns in the qualitative data across cases and sites, we will employ Qualitative Comparative Analysis (QCA) to enable decisive cross-case patterns to be identified, which is the usual domain of quantitative analysis. QCA respects the heterogeneity of the contexts and different causally relevant conditions by comparing cases as configurations. This method will help to systematise our qualitative analysis across four research sites, working with four researchers, 15 CLOs multiple participants and stakeholders. We will produce statements of the combination of contextual mechanisms, programme mechanisms, and agents' responses that lead to improvements in health and wellbeing outcomes. Where there are differences in outcomes, we will seek to identify the variations in configurations of mechanisms that lead to differing results.

This early work, together with input from our Lived Experience Panel, will be used to develop and refine programme theories. These will then be presented to stakeholders in interviews and finally in workshops with a view to further refining. These initial programme theories will be tested in phase 2.

## Phase 2: testing programme theories, economic analysis, sustainability, and scale

### *Questionnaire study*

A longitudinal questionnaire will generate data about new participants in CLOs focussing on resource use and outcomes at baseline, 1, 6 and 12 months. Questionnaires will be administered in person, by telephone or otherwise remotely (using MS Teams and REDCap software) to maximise response rates. For consistency and comparability, follow up questionnaires will adopt the same mode of administration as baseline, as far possible. Eligibility criteria for the questionnaire study are shown in Table 1.

Table 1: Eligibility Criteria for questionnaire study

| Inclusion criteria | Exclusion criteria |
|---|---|
| Aged over 18 years | Ongoing participation in multiple CLOs |
| Community participant associated with participating CLOs | One-time participation only |
| Participant in an activity that involves several contacts over time (CLOs might provide one-time advice services for example but, for comparability, we will focus on a period of consistent participation). | Not within the community of the CLO. |

For peer review only

Eligible participants who express interest in taking part in the study via the CLO staff will be contacted by researchers to provide study information, complete consent procedures and administer a baseline questionnaire comprised of sociodemographic questions, resource use questions and a number of standardised outcome measures.

The primary outcome measure for the questionnaire study is the ICECAP-A (ICEpop CAPability measure for adults[32]. The ICECAP-A has been selected for three important reasons. Firstly, the dimensions of the ICECAP-A map well onto the intermediate outcomes identified in previous empirical research and in practice-based models synthesised in our conceptual model (see Figure 3) and so we set out to measure things that have been shown to be important to beneficiaries, practitioners and according to theory. Secondly it is validated and is supported for use in economic evaluation by NICE[33] and thirdly preference-based population tariffs are available[34]. Our secondary outcome measures are health status using EQ-5D 5L[35], Warwick Edinburgh Mental Wellbeing Scale (WEMWBS)[36] , and Social Connectedness [37].

There is no estimate of minimal important difference available from existing datasets of ICECAP-A, so the study will be designed to detect a commonly used standardised effect size of 0.25 at 6 months. This represents a small to medium effect size. A sample size of 252 will be required to provide 80% power at 5% significance (with two-sided alpha) to detect a difference of 0.25 SDs. We will recruit 360 to achieve an effective sample of 252 completed questionnaires at 6 months, which allows for credible retention of 70%. We anticipate 60 to 65% retention at 12 months (n=225).

A statistical analysis plan will be agreed before database lock. Descriptive statistics will be used to explore and analyse the data for associations. Random effects regressions with clustered standard errors will be employed to account for the longitudinal nature of our data and for the fact individuals are clustered in CLOs. Multilevel modelling that explicitly acknowledges the hierarchical clustering of the data will be employed[38,39]. These models will estimate the impact of the CLOs on the outcome measures over time after controlling for individual and CLO characteristics. The analyses will be run for our primary and secondary outcomes separately.

### *Q Methodology study*

The aim of all Q studies is to identify patterns of shared perspectives[40–42]. In the context of realist methods this sits well with the goal of uncovering regularities in data[43]. We will make use of this approach to unpack the mechanisms at work in different contexts[44]. The Q set of statements will comprise of *candidate mechanisms* drawn from theory, interview and workshop data and hypotheses that emerge from preliminary analysis of context-outcome patterns in the questionnaire study.

Up to 60 participants, selected from the questionnaire sample to cover different contexts and outcomes, will complete Q sorts to identify which mechanisms they consider help to explain why outcomes arise (or do not) in context. Different mechanisms might relate to the same outcomes in different contexts and we will gather descriptive information about Q

For peer review only

participants' contexts. Factor analysis of Q sort data based on correlations between individuals' Q sorts will reveal shared patterns of context and mechanisms for interpretation.

### *Economic analysis*

The economic evaluation will take the form of a cost consequence analysis (CCA). A CCA presents costs and outcomes in a disaggregated form which is appropriate given the multi-sectoral context in which the CLOs operate and the range of outcomes of importance[45]. We will seek to quantify resource implications arising from both contextual (CM) and programme mechanisms (PM), and how these relate to agents' responses (A) and outcomes (O) measured.

The first stage of the economic analysis will be to map out the resource inputs required to deliver the activities provided by the CLOs. This will be done in collaboration with the CLOs and will be assessed and updated throughout the project. All resource inputs will be totalled and turned into a cost per participant. Secondly, as part of the questionnaire study, participants will self-report use of other health care, social and community-based services in the month prior to joining the CLO and then at 1, 6 and 12-month follow up. This will allow us to assess changes in resource use within the study participant group in the form of a before-and-after design. Units of each item will be recorded and presented along with unit costs deriving from local publicly available sources[46].

The resource use will be presented alongside the estimates of health and wellbeing outcomes from the primary and secondary outcome measures and with qualitative data from the interviews conducted in both phase 1 and 2 of the study in the CCA.

### *Sustainability and scalability*

Building on information deriving from our economic analysis and relevant financial documents (e.g. contracts and agreements) provided by CLOs managers, our assessment of financial sustainability and scalability of CLOs will be informed by interviews with CLO stakeholders (e.g. managers, funders). Combining economic evaluation with qualitative interview data will help to better understand *processes* associated with developing sustainable CLOs. The interviewees will be asked about best practices and key challenges involved in establishing, operating and growing sustainable CLOs, and what it means to be 'sustainable' in their specific community contexts. Previous research[47] suggests that sustainability and scalability in a community context is rarely straightforward and CLOs frequently face difficulties in maintaining and sustaining their services and activities.

Drawing on a sample of diverse stakeholders from a range of CLOs (i.e. variation across rural/urban, organisational size and maturity, range of activities etc.), will help us to explore how different contexts can promote or inhibit particular mechanisms; intended and unintended outputs and outcomes; income streams and views about sustainability.

For peer review only

## Patient and Public Involvement

Public involvement in the preparation and design of the project as presented to funders was achieved through co-investigators and collaborators in the community sector who work in and with CLOs and are co-authors on this paper.

Patient and public involvement and engagement (PPIE) has been integrated throughout the project by establishing a panel of individuals with experience of living in our CLO communities. The 'Lived Experience Panel' (LE Panel) will ensure that the project is informed and guided by ongoing community expertise, perspective and voice and that findings are relevant and meaningful to community organisations.

The LE Panel will comprise representatives of the CLOs, identified and recruited with the support of our community partners. The LE Panel will meet at key stages of the research process to guide and inform the research questions, methods, data analysis and interpretation of results – with space for ongoing involvement and communication between Panel meetings. The hands-on nature of these participatory methods and the support of the research team will allow participants to actively engage with the research, placing more value on their expertise. Representatives of the LE Panel will also be members of the Study Steering Committee, complementing the perspectives of other key stakeholders.

Training opportunities will be provided allowing participants to build their skills and knowledge around community-based research, promoting two-way learning exchange between the researchers and Panel participants, which will elevate community voices and strengthen researcher-participant relations. Continuous evaluation will be fostered to improve facilitation and promote learning throughout the life and work of the Panel

## Ethics and dissemination

This project has been approved by the Glasgow Caledonian University School of Health and Life Sciences Ethics Committee on 15/7/21 (ref HLS/NCH/20/034) and subsequently affirmed by Ethics Committees at Bournemouth University, Queens University Belfast (MHLS 21_94) and University of East London (ETH2021-0226). Common Health Assets does not involve any NHS sites, staff or patients. IRAS and NHS REC are not required. The project is funded for three years beginning 1st September 2021.

This project will advance the research field, at the nexus of public health research and community development/social enterprise research and methodologically in terms of economics and quantitative approaches in realist evaluation and in the use of Q methodology to explore mechanisms. Our PPIE strategy including lived experience panel, stakeholder involvement in the research team and Study Steering Committee (SSC), and policy briefing will ensure that the research involves users of evidence throughout to enhance the relevance and application to policy and practice. CLOs will be able to use research findings as part of their impact evidence base when applying for public funding or in refining their activities where appropriate. We will disseminate our findings using a range of media, and pitched to suit the needs of our three main constituencies of interest; namely: (i) Practitioners and activists; (ii) Policymakers and Commissioners; and (iii) Academics.

For peer review only - http://bmjopen.bmj.com/site/about/guidelines.xhtml

The project outputs will comprise peer reviewed papers in academic journals, blogs, short videos, photography exhibition, policy briefing papers, website ([www.commonhealthassets.uk](http://www.commonhealthassets.uk)) and a commissioning guide. We will hold workshops to share findings and develop recommendations with practitioners and policy actors. Each CLO will receive a report summarising the study findings and their own local findings where possible.

## Authors' contributions

This paper is based on a longer protocol approved by the NIHR and drafted by all co-investigators. Rachel Baker led on the design of the research and the writing of the protocol, producing the initial draft. All authors revised subsequent drafts and agreed final text.

## Funding

This study is funded by the National Institute for Health Research (NIHR) Public Health Research programme (NIHR 129118]. The views expressed are those of the author(s) and not necessarily those of the NIHR or the Department of Health and Social Care.

## Conflicts of interests statement

None of the authors have a financial interest in the results of this research.

## Data Statement

We intend to publish our data to a recognised UK data repository if it is practically possible to do. Data sharing statements will be included when publishing the findings of this research which will describe how to access the data. The sharing of data will be done in the most appropriate way and only if it is clear that sharing will help deliver research that maximises benefits to the public. Final decisions with respect to data sharing will be taken with the following in mind: protect the confidentiality and privacy of individuals; respect the terms of consent by individuals who are involved in the study; be consistent with relevant legal, ethical and regulatory frameworks; and guard against unreasonable resource use to share the data. Researchers and other users will have to apply to gain access to the data and the process of gaining access will be governed by Glasgow Caledonian University and NIHR data sharing guidelines.

## Acknowledgements

The proposed research would not be possible without the partnership of our community led organisations and their community participants and volunteers, without whom the research would be impossible.  Their commitment and collaboration are particularly welcome given the Covid-19 pandemic, during which we planned to start this work and when CLOs were supporting their communities.

For peer review only

# References


1. Blas E, Gilson L, Kelly MP, et al. Addressing Social Determinants of Health Inequities: What Can the State and Civil Society Do? *The Lancet*. 2008;372(9650):1684-1689.

2. Garven F, Pattoni L, McLean J. *Asset-Based Approaches: Their Rise, Role and Reality*. Dunedin Academic Press; 2016.

3. Roy MJ. The Assets-Based Approach: Furthering a Neoliberal Agenda or Rediscovering the Old Public Health? A Critical Examination of Practitioner Discourses. *Crit Public Health*. 2017;27(4):455-464.

4. NHS England. *Five Year Forward View*. NHS England; 2014. https://www.england.nhs.uk/wp-content/uploads/2014/10/5yfv-web.pdf

5. VCSE Sector, Department of Health, NHS England, Public Health England. *The Joint VCSE Review Action Plan*. Department of Health; 2018. Accessed April 4, 2019. https://voluntarycommunitysocialenterprisereview.files.wordpress.com/2019/03/joint-vcse-review-action-plan-2018.pdf

6. Public Health England. *A Guide to Community-Centred Approaches for Health and Wellbeing*. Public health England; 2015. Accessed July 9, 2019. https://assets.publishing.service.gov.uk/government/uploads/system/uploads/attachment_data/file/768979/A_guide_to_community-centred_approaches_for_health_and_wellbeing__full_report_.pdf

7. Renewing Scotland's public services: priorities for reform in response to the Christie Commission. Accessed September 28, 2022. http://www.gov.scot/publications/renewing-scotlands-public-services-priorities-reform-response-christie-commission/

8. Van Bortel T, Wickramasinghe ND, Morgan A, Martin S. Health assets in a global context: a systematic review of the literature. *BMJ Open*. 2019;9(2):e023810.

9. McLean J, McNeice V, Mitchell C. *Asset-Based Approaches in Service Setting: Striking a Balance*. Glasgow Centre for Population Health; 2017. Accessed July 24, 2019. https://www.gcph.co.uk/assets/0000/6049/Asset_based_approaches_in_service_settings.pdf

10. Glasgow Centre for Population Health, Scottish Community Development Centre. *Positive Conversations, Meaningful Change: Learning from Animating Assets*.; 2015. Accessed September 22, 2022. https://www.gcph.co.uk/assets/0000/5433/0356_learning_from_animating_assets_web.pdf

11. Fisher B. *Community Development in Health: A Literature Review*.; 2016.


For peer review only

12. Tailor-made: how community groups improve people's lives. Trust for London. Accessed September 22, 2022. https://www.trustforlondon.org.uk/publications/tailor-made-how-community-groups-improve-peoples-lives/

13. Skivington K, Matthews L, Simpson SA, et al. A new framework for developing and evaluating complex interventions: update of Medical Research Council guidance. *BMJ*. 2021;374:n2061.

14. Kelly D, Steiner A, Mazzei M, Baker R. Filling a void? The role of social enterprise in addressing social isolation and loneliness in rural communities. *J Rural Stud*. 2019;70:225-236.

15. Calo F, Roy MJ, Donaldson C, Teasdale S, Baglioni S. Exploring the contribution of social enterprise to health and social care: A realist evaluation. *Soc Sci Med*. 2019;222:154-161. doi:10.1016/j.socscimed.2019.01.007

16. Macaulay B, Mazzei M, Roy MJ, Teasdale S, Donaldson C. Differentiating the effect of social enterprise activities on health. *Soc Sci Med*. 2018;200:211-217.

17. Roy MJ, Baker R, Kerr S. Conceptualising the Public Health Role of Actors Operating Outside of Formal Health Systems: The Case of Social Enterprise. *Soc Sci Med*. 2017;172:144-152. doi:10.1016/j.socscimed.2016.11.009

18. Pawson R, Tilley N. *Realistic Evaluation*. Sage; 1997.

19. Porter S. Realist Evaluation: An Immanent Critique. *Nurs Philos*. 2015;16(4):239-251.

20. English indices of deprivation 2019: mapping resources. GOV.UK. Accessed September 28, 2022. https://www.gov.uk/guidance/english-indices-of-deprivation-2019-mapping-resources

21. Scottish Index of Multiple Deprivation 2020. Accessed September 28, 2022. https://www.gov.scot/collections/scottish-index-of-multiple-deprivation-2020/

22. Northern Ireland Multiple Deprivation Measure 2017 (NIMDM2017). Northern Ireland Statistics and Research Agency. Accessed September 28, 2022. https://www.nisra.gov.uk/statistics/deprivation/northern-ireland-multiple-deprivation-measure-2017-nimdm2017

23. Macaulay B, Roy MJ, Donaldson C, Teasdale S, Kay A. Conceptualizing the Health and Well-Being Impacts of Social Enterprise: A UK-Based Study. *Health Promot Int*. 2018;33(5):748-759.

24. Morgan A, Davies M, Ziglio E, eds. *Health Assets in a Global Context: Theory, Methods, Action: Investing in Assets of Individuals, Communities and Organizations*. Springer; 2010.

25. Morgan A, Ziglio E. Revitalising the Evidence Base for Public Health: An Assets Model. *Glob Health Promot*. 2007;14(2 suppl):17-22.


For peer review only

26. Calò F, Roy MJ, Donaldson C, Teasdale S, Baglioni S. Exploring the Contribution of Social Enterprise to Health and Social Care: Approaches and Considerations. *Soc Enterp J*. 2021;17(1):140-155.

27. Munford LA, Sidaway M, Blakemore A, Sutton M, Bower P. Associations of Participation in Community Assets with Health-Related Quality of Life and Healthcare Usage: A Cross-Sectional Study of Older People in the Community. *BMJ Open*. 2017;7(2):e012374.

28. Bellazzecca E, Baker R, Bertotti M, et al. CommonHealth Assets: a protocol for a realist review of reviews of the health and wellbeing impact of community led organisations in disadvantaged communities. PROSPERO 2021 CRD42021287959. Published online October 29, 2021. Accessed September 28, 2022. https://www.crd.york.ac.uk/prospero/display_record.php?RecordID=287959

29. Wang Caroline BMA. Photovoice: Concept, Methodology, and Use for Participatory Needs Assessment. *Health Educ Behav*. 1997;24(3):369-387.

30. QSR International Pty Ltd. NVivo. Published online 2020. https://www.qsrinternational.com/nvivo-qualitative-data-analysis-software/home?_ga=2.78631316.1092956994.1664355022-573106870.1664355022

31. Roller MR, Lavrakas PJ. *Applied Qualitative Research Design: A Total Quality Framework Approach*. The Guilford Press; 2015:xviii, 398.

32. Al-Janabi H, N Flynn T, Coast J. Development of a self-report measure of capability wellbeing for adults: the ICECAP-A. *Qual Life Res*. 2012;21(1):167-176.

33. 7 Incorporating economic evaluation | The social care guidance manual | Guidance | NICE. Accessed September 28, 2022. https://www.nice.org.uk/process/pmg10/chapter/incorporating-economic-evaluation

34. Flynn TN, Huynh E, Peters TJ, et al. Scoring the Icecap-a capability instrument. Estimation of a UK general population tariff. *Health Econ*. 2015;24(3):258-269.

35. EuroQol Group. EuroQol--a new facility for the measurement of health-related quality of life. *Health Policy Amst Neth*. 1990;16(3):199-208.

36. Tennant R, Hiller L, Fishwick R, et al. The Warwick-Edinburgh Mental Well-being Scale (WEMWBS): Development and UK validation. *Health Qual Life Outcomes*. 2007;5:No Pagination Specified-No Pagination Specified.

37. Lee RM, Robbins SB. Measuring belongingness: The Social Connectedness and the Social Assurance scales. *J Couns Psychol*. 1995;42:232-241.

38. Goldstein H. *Multilevel Statistical Models*. John Wiley & Sons; 2011.

39. Steele F. Multilevel models for longitudinal data. *J R Stat Soc Ser A Stat Soc*. 2008;171(1):5-19.

For peer review only

40. Baker R, Thompson C, Mannion R. Q methodology in health economics. *J Health Serv Res Policy*. 2006;11(1):38-45.

41. Watts S, Stenner P. *Doing Q Methodological Research; Theory Method and Interpretation*. SAGE Publications Ltd; 2012.

42. Van Exel J, De Graaf G. Q methodology: A sneak preview. *Retrieved January*. 2005;24. Accessed August 31, 2017. https://www.researchgate.net/profile/Gjalt_Graaf/publication/228574836_Q_Methodology_A_Sneak_Preview/links/02bfe50f946fc9978b000000.pdf

43. Duncan Millar J, Mason H, Kidd L, IMPETUS Research Team. What is important in supporting self-management in community stroke rehabilitation? A Q methodology study. *Disabil Rehabil*. Published online June 25, 2022:1-9.

44. Astbury B. Making Claims Using Realist Methods. In: Emmel N, Greenhalgh J, Manzano A, Monaghan M, Dalkin S, eds. *Doing Realist Research*. SAGE Publications Ltd; 2018:59-78.

45. Edwards RT, McIntosh E, eds. *Applied Health Economics for Public Health Practice and Research*. Oxford University Press; 2019.

46. Curtis CL, Burns A. Unit Costs of Health and Social Care 2018. Published online 2018:203.

47. Lyon F, Fernandez H. Strategies for scaling up social enterprise: lessons from early years providers. *Soc Enterp J*. 2012;8(1):63-77.

For peer review only

**Figure 1 – Study illustration: phase 1 and phase 2 methods and outputs**

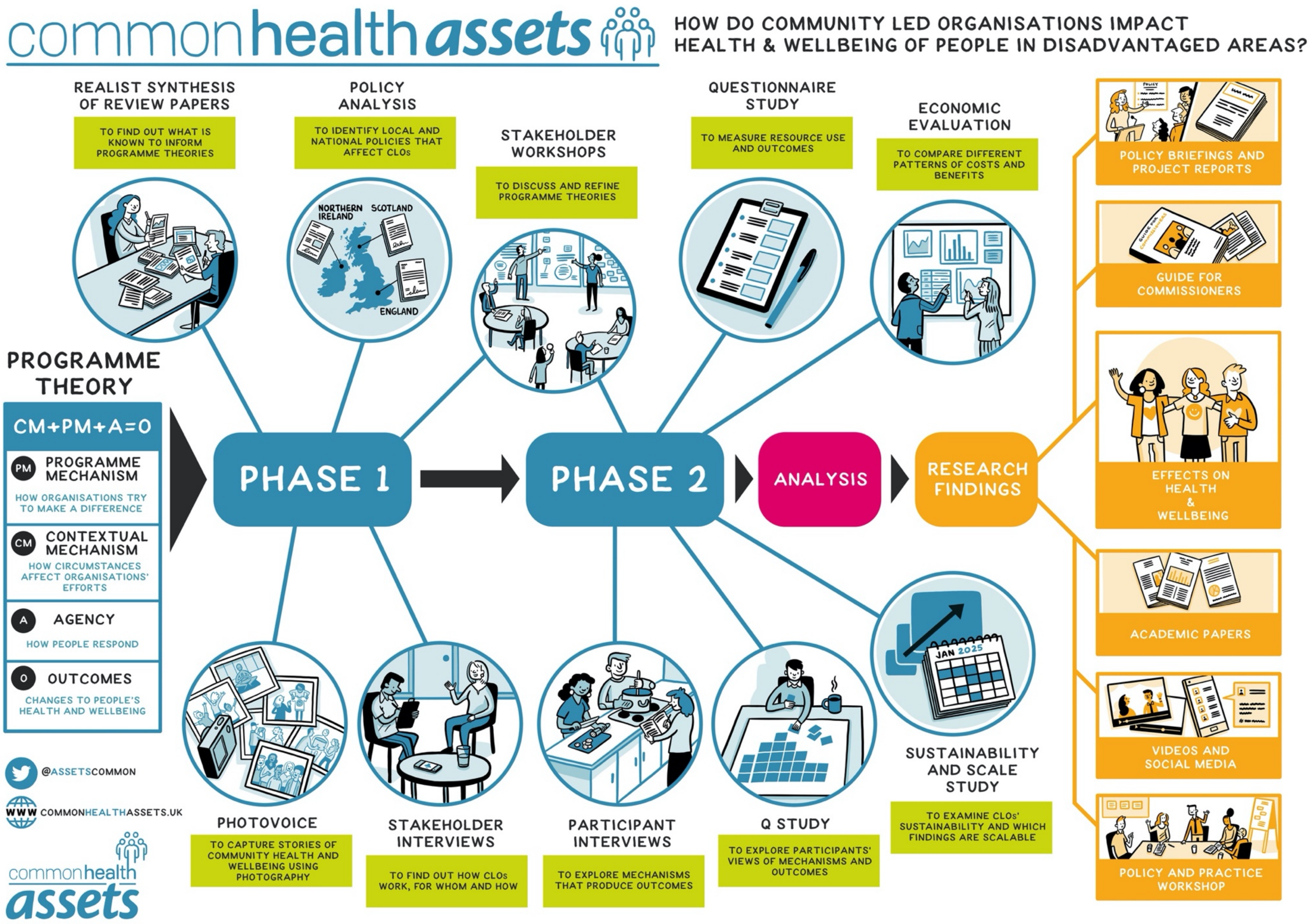

FIGURE 2–'PLUMBING DIAGRAM' SHOWING HOW DATA SOURCES FEED INTO THE REALIST FRAMEWORK CONNECTING CONTEXT, MECHANISMS AND OUTCOMES.

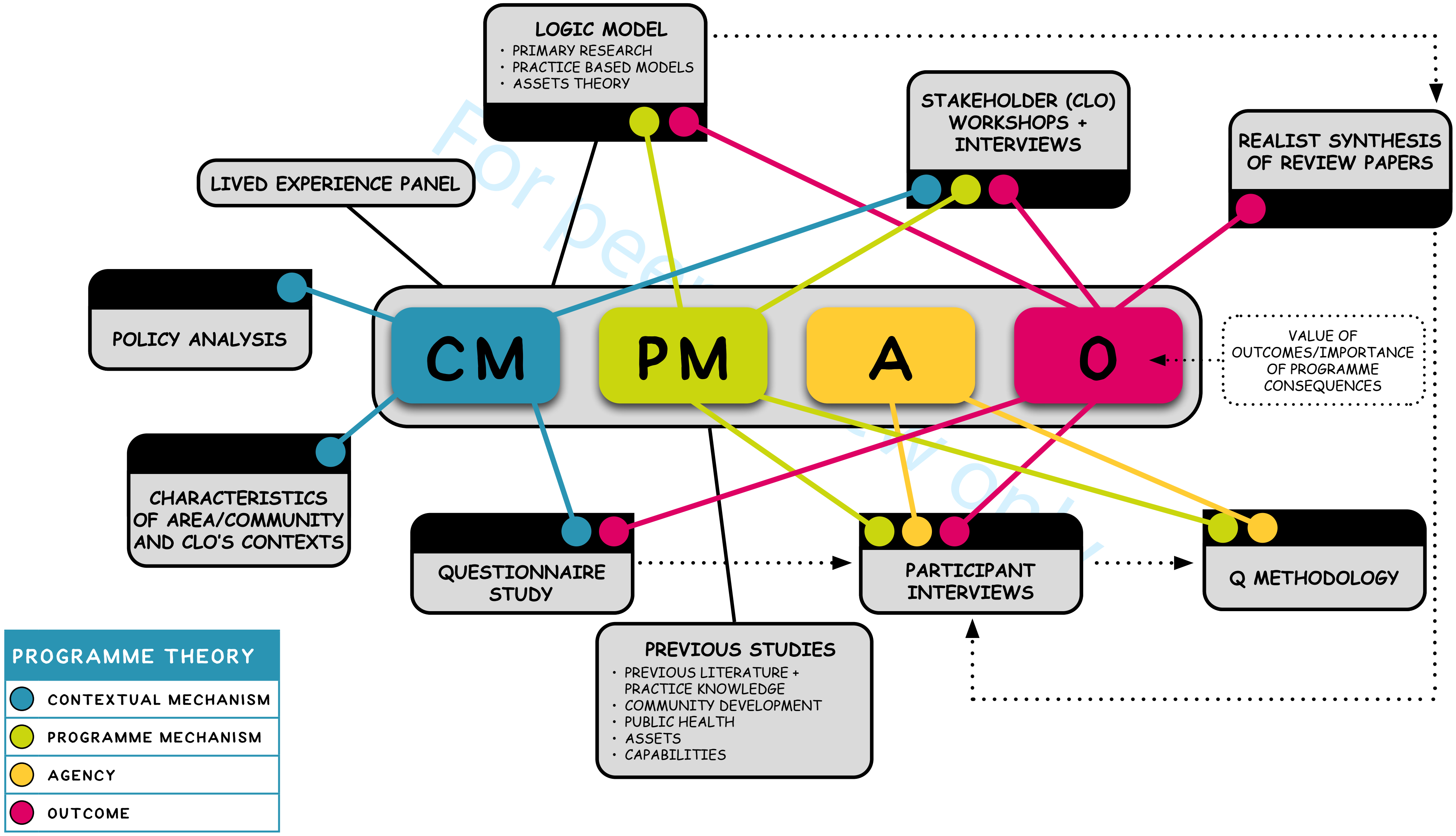


For peer review only - http://bmjopen.bmj.com/site/about/guidelines.xhtml

**REFERENCE:** PORTER, S. (2015) REALIST EVALUATION: AN IMMANENT CRITIQUE, NURSING PHILOSOPHY, 16, 239–251

FIGURE 3 CONCEPTUAL MODEL – A SYNTHESIS OF RESEARCH AND PRACTICE-BASED MODELS.

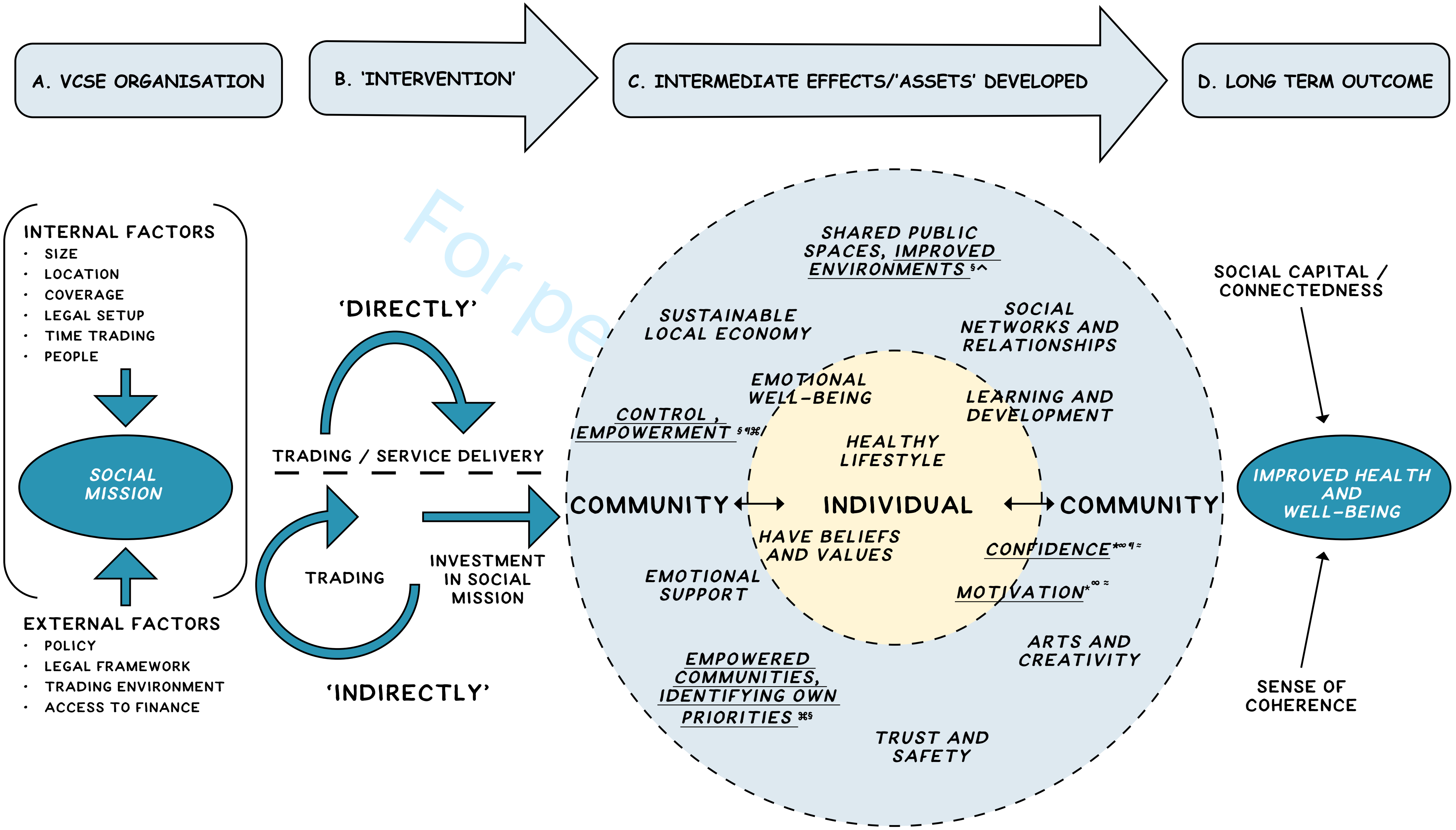


For pe

**KEY TO MODEL WITH ADDITIONS TO COMMONHEALTH CONCEPT MODEL, FOLLOWING SYNTHESIS UNDERLINED:**
CALO ET AL (2018); ∞KELLY ET AL (2019); §MACAULEY ET AL (2017); ≈ROY ET AL (2017); ^HEALTH ASSETS MODEL (MORGAN ET AL 2007, 2010)
¶NNHSA MODEL OF HEALTH CREATION; ⌘SCDC COMMUNITY LED HEALTH MODEL

BMJ Open

# Common Health Assets protocol: a mixed-methods, realist evaluation and economic appraisal of how community-led organisations (CLOs) impact on the health and wellbeing of people living in deprived areas

| | |
|---|---|
| Journal: | *BMJ Open* |
| Manuscript ID | bmjopen-2022-069979.R1 |
| Article Type: | Protocol |
| Date Submitted by the Author: | 10-Jan-2023 |
| Complete List of Authors: | Baker, Rachel; Glasgow Caledonian University, Yunus Centre for Social Business and Health<br>Ahmed, Mohasin; Glasgow Centre for Population Health<br>Bertotti, Marcello; University of East London, Institute for Connected Communities<br>Cassidy, John; Scottish Communities for Health and Wellbeing<br>Chipuriro, Rejoice; Bournemouth University<br>Clewett, Emma; University of East London<br>Donaldson, Cam; Glasgow Caledonian University, Yunus Centre for Social Business and Health<br>Elders, A; Glasgow Caledonian University School of Health and Life Sciences, NMAHP Research Unit ; Glasgow Caledonian University<br>Fenge, Lee Ann; Bournemouth University<br>Fox, Julie; Annexe Communities Glasgow<br>Galway, K.; Queen's University Belfast<br>Gildea, Aideen; Queen's University Belfast<br>McGuiness, Aileen; Bogside and Brandywell Health Forum<br>McLean, Jennifer; Glasgow Centre for Population Health<br>Manoukian, Sarkis; Glasgow Caledonian University, Yunus Centre for Social Business and Health<br>Mason, Helen; Glasgow Caledonian University, Yunus Centre for Social Business and Health<br>Morgan, A; Glasgow Caledonian University London, Yunus Centre for Social Business and Health<br>Mulholland, Jill; Queen's University Belfast<br>O'Hare, Liam; Queens University of Belfast<br>Paterson, Andrew; Scottish Community Development Centre<br>Porter, Sam; Bournemouth University, Social Sciences and Social Work<br>Rendall, Jack; Glasgow Caledonian University, Yunus Centre for Social Business and Health<br>Roy, Michael; Glasgow Caledonian University,<br>Seaman, Peter; Glasgow Centre for Population Health<br>Simpson, Merron; The Health Creation Alliance<br>Steiner, Artur; Glasgow Caledonian University, Yunus Centre for Social Business and Health<br>Kelly, Michael; University of Cambridge, Public Health and Primary Care |
| <b>Primary Subject | Public health |

| | |
|---|---|
| Heading</b>: | |
| Secondary Subject Heading: | Qualitative research, Health economics |
| Keywords: | PUBLIC HEALTH, SOCIAL MEDICINE, HEALTH ECONOMICS |
| | |



SCHOLARONE™
Manuscripts

## Title

Common Health Assets protocol: a mixed-methods, realist evaluation and economic appraisal of how community-led organisations (CLOs) impact on the health and wellbeing of people living in deprived areas

## Corresponding Author

Professor Rachel Baker PhD
Yunus Centre for Social Business and Health
Glasgow Caledonian University
70 Cowcaddens Road
Glasgow G4 0BA
Rachel.Baker@gcu.ac.uk
Tel: 0141 331 8246
Fax: n/a

## Co Authors

Miss Mohasin Ahmed, Glasgow Centre for Population Health, Mohasin.Ahmed@glasgow.ac.uk (Glasgow, Scotland)
Dr Marcello Bertotti PhD, Institute for Connected Communities, University of East London, m.bertotti@uel.ac.uk (London, England)
Dr John Cassidy, Scottish Communities for Health and Wellbeing, john.kcassidy@ntlworld.com (Glasgow, Scotland)
Dr Rejoice Chipuriro PhD, Institute for Connected Communities, Bournemouth University, rchipuriro@bournemouth.ac.uk (Bournemouth, England)
Miss Emma Clewett, University of East London, eclewett@uel.ac.uk (London, England)
Professor Cam Donaldson PhD FRSE, Yunus Centre for Social Business and Health, GCU, Cam.Donaldson@gcu.ac.uk (Glasgow, Scotland)
Dr Andrew Elders, NMAHP Research Unit, GCU Andrew.Elders@gcu.ac.uk (Glasgow, Scotland)
Professor Lee Ann Fenge, Bournemouth University, lfenge@bournemouth.ac.uk (Bournemouth, England)
Ms Julie Fox, Annexe Communities Glasgow, Julie.fox@annexecommunities.org.uk (Glasgow, Scotland)
Dr Karen Galway, Queen's University Belfast, k.galway@qub.ac.uk (Belfast, Northern Ireland)
Dr Aideen Gildea, Queen's University Belfast, a.gildea@qub.ac.uk (Belfast, Northern Ireland)
Miss Aileen McGuiness, Bogside and Brandywell Health Forum Derry, aileen@bbhealthforum.org (Derry, Northern Ireland)
Dr Jennifer McLean PhD, Glasgow Centre for Population Health, jennifer.mclean@glasgow.ac.uk (Glasgow, Scotland)
Dr Sarkis Manoukian PhD, Yunus Centre for Social Business and Health, GCU Sarkis.Manoukian@gcu.ac.uk (Glasgow, Scotland)
Professor Helen Mason PhD, Yunus Centre for Social Business and Health, GCU Helen.Mason@gcu.ac.uk (Glasgow, Scotland)
Professor Antony Morgan PhD, Yunus Centre, GCU London, Antony.Morgan@gcu.ac.uk (London, England)
Ms Jill Mulholland, Queen's University Belfast, jmulholland18@qub.ac.uk (Belfast, Northern Ireland)

For peer review only

Dr Liam O'Hare, School of Social Sciences, Education and Social Work, Queen's University Belfast, L.OHare@qub.ac.uk (Belfast, Northern Ireland)
Dr Andrew Paterson, Scottish Community Development Centre, andrew.paterson@scdc.org.uk (Glasgow, Scotland)
Professor Sam Porter, Bournemouth University, porters@bournemouth.ac.uk (Bournemouth, England)
Dr Jack Rendall PhD, Yunus Centre for Social Business and Health, GCU Jack.Rendall@gcu.ac.uk (Glasgow, Scotland)
Professor Michael J Roy PhD, Yunus Centre for Social Business and Health, GCU Michael.Roy@gcu.ac.uk (Glasgow, Scotland)
Dr Peter Seaman PhD, Glasgow Centre for Population Health, peter.seaman@glasgow.ac.uk (Glasgow, Scotland)
Ms Merron Simpson, Health Creation Alliance, merron@thehealthcreationalliance.org (Birmingham, England)
Professor Artur Steiner PhD, Yunus Centre for Social Business and Health, GCU Artur.Steiner@gcu.ac.uk (Glasgow, Scotland)
Professor Michael P Kelly, University of Cambridge, mk744@medschl.cam.ac.uk (Edinburgh, Scotland)

For peer review only

## Abstract

### Introduction

This research investigates how community-led organisations' (CLOs') use of *assets-based approaches* improves health and wellbeing, and how that might be different in different contexts. Assets based approaches involve 'doing with' rather than 'doing to' and bring people in communities together to achieve positive change using their own knowledge, skills, and experience. Some studies have shown that such approaches can have a positive effect on health and wellbeing. However, research is limited, and we know little about which approaches lead to which outcomes, and how different contexts might affect success.

### Methods and analysis

Using a realist approach, we will work with 15 CLOs based in disadvantaged communities in England, Scotland, and Northern Ireland. A realist synthesis of review papers, and a policy analysis in different contexts, precedes qualitative interviews and workshops with stakeholders, to find out how CLOs' programmes work, and identify existing data. We will explore participants' experiences through: a Q methodology study; participatory photography workshops; qualitative interviews; and measure outcomes using a longitudinal survey, with 225 CLO participants, to assess impact for people who connect with the CLOs. An economic analysis will estimate costs and benefits to participants, for different contexts and mechanisms. A 'Lived Experience Panel' of people connected with our CLOs as participants or volunteers, will ensure the appropriateness of the research, interpretation and reporting of findings.

### Ethics and dissemination

This project, research tools and consent processes have been approved by the Glasgow Caledonian University School of Health and Life Sciences Ethics Committee, and affirmed by

Ethics Committees at Bournemouth University, Queen's University Belfast, and the University of East London. Common Health Assets does not involve any NHS sites, staff, or patients. Findings will be presented through social media, project website, blogs, policy briefings, journal articles, conferences, and visually in short digital stories, and photographic exhibitions.

## Abstract word count

341

## Keywords

Community development, community resources, evaluation study, quality of life, social determinants of health

## Word count

3745

## Article Summary

## Strengths and Limitations of this study

- Using a realist approach means that this study will build explicitly on theory and practice to generate understanding of what works, for whom, how and in what circumstances.
- A lived experience panel with input into design by community partners and stakeholders means that methods can be adjusted and improved in real time.
- Use of mixed methods will provide insights from different perspectives and test programme theories quantitatively as well as qualitatively.
- Our combination of realist and economic evaluation techniques will contribute towards knowledge in an area of ongoing methodological innovation.

## Introduction

This research focusses on place-based, community-owned organisations working in disadvantaged areas, which we label Community-Led Organisations (CLOs). CLOs have a critical role in the delivery of health and social care, tackling health inequalities and underlying social determinants of health; for example reducing loneliness and isolation, and increasing individual and community capacity for democratic participation[1]. CLOs work in ways that have come to be known as an assets-based approaches, which involve *doing with* rather than *doing to*, and bring people in communities together to achieve positive change using their own knowledge, skills, and experience. In the last decade there has been an emergence of terminology around assets-based approaches in community development and health. The premise is that sustained positive health and social outcomes occur when people and communities have opportunities and facilities to manage their own futures[2].

For peer review only

CLOs in the VCSE (voluntary, community, and social enterprise) sector[3] work in partnership with other organisations and networks, local government or health professionals. They are part of a developing field of collaborative public health initiatives that have received sustained policy attention[4–7] but evidence of their effectiveness remains limited. A recent systematic review[8] found that there is a variety of definitions of assets-based approaches, and that evidence in relation to health effects is scarce, largely presented in grey literature[9,10] with a predominance of case studies. This is replicated by reviews of community development and health literature,[11,12] which have observed that experimental approaches and controlled trials are often challenging when controlling 'exposure' is difficult and 'interventions' are complex[13]. Despite this lack of robust evidence, previous research on the work of the voluntary, community and social enterprise (VCSE) sector (e.g. the CommonHealth research programme[14–17]) provides a solid base for rigorous, theory-based evaluation to explore the contexts and mechanisms through which community-led approaches lead to improvements in health and wellbeing outcomes.

The Common Health Assets project will investigate the impact of community-led approaches on health and wellbeing in areas facing significant health and social inequalities, using a realist approach to determine 'what works, for whom, and in what circumstances'[18].

The project addresses the following research objectives:

1. To develop, with stakeholders, initial realist programme theories, to explain 'what works, for whom in what circumstances and how' in relation to CLOs' impact on health and wellbeing and health inequalities
2. To test and refine programme theories by locating existing data and generating mixed method evidence to identify context-mechanism-outcome configurations (CMOCs)
3. To estimate the resource use and outcomes associated with different CMOCs in an economic appraisal
4. To analyse CLO income streams and stakeholders' views on sustainability and scalability

## Methods and Analysis

### Study Design

The overarching methodology for this study is realist evaluation. Realist evaluation is a theory driven form of evaluation which recognises that not every intervention will work for each person in the same way[18]. Realist texts refer to the identification of context-mechanism-outcome configurations (CMOCs) as programme theories which can be tested and refined. Following Porter[19], we distinguish contextual mechanisms (CMs), which relate to factors within the social and organisational environment that influence people's attitudes and behaviour, from programme mechanisms (PM), which are the factors embedded in particular interventions designed to alter people's attitudes and behaviour. We include agency (A), which is the capacity of actors to interpret, evaluate and respond to external social influences. The combination of contextual, programme and agential mechanisms produces outcomes

For peer review only

(O). Outcomes of interest include both changes in behaviour over time, and effects on the flourishing or suffering of those involved.

This multi-method, realist study is designed in two phases as shown in Figure 1 – Study Illustration. In the first phase (employing realist synthesis, participatory photography workshops, and qualitative methods) we will develop and refine programme theories describing CLOs' impact on health and wellbeing, focussing on the contextual and programme mechanisms that affect outcomes in different ways for different groups of people. In the second phase we will test the programme theories by: using a longitudinal participant survey, participant interviews and Q-sort methods; examining the economic implications of findings in terms of the relationship between resources and outcomes of a range of programme theories; and exploration of sustainability and scalability of CLOs.

****Insert figure one around here****

Figure 2 is a 'Plumbing Diagram' showing the contribution of different sources of information and how the various study components and data sources contribute to our understanding of contextual and programme mechanisms, actors' responses and outcomes, and the evolving programme theory.

****Insert figure two around here****

## Study Setting and participants

The project will be conducted in Scotland, Northern Ireland, and England. We will work with CLOs in communities with multiple disadvantages. This will be defined here as CLOs working with communities, and with people, in Index of Multiple Deprivation (IMD) deciles 1-3: that is, 10% to 30% most deprived, based on the IMDs for each country[20–22]. A researcher will be appointed in each area to work closely with CLOs. Approximately 15 CLOs will be recruited to the study. Although CLOs are similar in their focus on disadvantaged communities and in many of their approaches, we will select CLOs as research partners with the aim of achieving variation over range of attributes including: geography and demography; CLO size and funding sources; characteristics of participant populations; premises or other physical assets; number of volunteers and professionals; range of activities available; relationships with local government, social and health services.

Recruitment of participants for all study components will be through the 15 CLOs involved in the research. For qualitative methods (see Figures 1 and 2), including interviews, workshops, participatory photography and storytelling workshops, and Q sorting, sampling will be initially driven to achieve diversity through maximum variation, and subsequently more purposive – identifying key participants who might shine light on programme theories or offer different perspectives. Sampling for the survey will identify participants in CLO activities based on the inclusion and exclusion criteria outlined below and invite them to take part in the research by completing questionnaires at baseline and follow up, as described under phase 2 below.

For peer review only

## Phase 1: developing and refining programme theories

Our starting point is represented by a conceptual model that was developed based on a the 'CommonHealth' programme of research (http://www.commonhealth.uk/). This has been augmented with findings from qualitative research[14,23]; assets theory [3,24,25]; and practice-based models of health creation and community development[10,26,27] (Figure 3 Conceptual Model). The model sets out the potential pathways of effect, from CLOs' activities through to changes in health and wellbeing. It does *not* set out the ways in which impacts are realised or how contextual or programme mechanisms might lead to different agents' responses and outcomes. From this starting point we will gather information from a number of sources to propose and refine programme theories and set out the contextual and programme mechanisms likely to impact on actors and outcomes.

****Insert figure three around here****

A **realist synthesis of literature reviews** (peer reviewed and grey literature) will extract potential contextual factors, programme mechanisms and outcomes. Further details on the realist synthesis are provided in PROSPERO[28].

**Policy analysis** will draw on policy documents identified as relevant in the contexts in which the CLOs are embedded. Interviews and focus groups with CLO staff will explore how the policy and funding contexts differ in each setting, and how this potentially impacts on CLOs and on outcomes. A purposive sample will be selected from CLO staff at different level of the organisation and other key informants with influence on the work of CLOs. The analysis will focus on actions and activities that are policy-driven or -contingent (including barriers to action and unintended consequences of policies) to reveal potential (outer) contextual mechanisms that might inhibit or enhance outcomes. The nature of relationships between the local public sector and CLOs will be explored, for instance: differences between policy rhetoric and reality; the availability and stability of funding; whether different attitudes exist towards CLOs to address local social vulnerabilities in each of the different contexts; and whether such differences matter.

**Participatory photography workshops** with CLO participants will focus on contextual mechanisms and how communities and CLOs create the conditions or barriers to health and wellbeing[29] (See www.photovoice.org) . These workshops will elicit community participants' views of what creates or prevents good health and wellbeing in their communities. These workshops provide community members with the opportunity to learn about photography and create images that represent, within their communities, those things that contribute to or detract from their wellbeing, and tell a story about what each image means.

**Stakeholder interviews and workshops** will build on the realist review and policy analysis, to explore the design of programmes in CLOs, and what works, for whom and how, to produce health and wellbeing outcomes. In Phase 1 we will engage with 40-60 stakeholders in interviews and workshops.

For peer review only - http://bmjopen.bmj.com/site/about/guidelines.xhtml

Qualitative data will be coded in terms of Contextual Mechanisms, Programme Mechanisms, Agency and Outcomes. Initial programme theories will be refined by referring to codes that index data in this way, always focusing on what works, for whom, in what contexts. Importantly there will be unintended outcomes, positive and negative, and data will be searched for confirmatory as well as divergent patterns. Qualitative data will be transcribed verbatim and coded using NVivo[30] initially using broad 'bucket coding' to identify themes in the data[31] and thereafter in relation to programme theories.

As programme theories are refined, and with a view to exploring patterns in the qualitative data across cases and sites, we will employ Qualitative Comparative Analysis (QCA) to enable decisive cross-case patterns to be identified, which is the usual domain of quantitative analysis. QCA respects the heterogeneity of the contexts and different causally relevant conditions by comparing cases as configurations. This method will help to systematise our qualitative analysis across four research sites, working with four researchers, 15 CLOs multiple participants and stakeholders. We will produce statements of the combination of contextual mechanisms, programme mechanisms, and agents' responses that lead to improvements in health and wellbeing outcomes. Where there are differences in outcomes, we will seek to identify the variations in configurations of mechanisms that lead to differing results.

This early work, together with input from our Lived Experience Panel, will be used to develop and refine programme theories. These will then be presented to stakeholders in interviews and finally in workshops with a view to further refining. These initial programme theories will be tested in phase 2.

## Phase 2: testing programme theories, economic analysis, sustainability, and scale

### *Questionnaire study*

A longitudinal questionnaire will generate data about new participants in CLOs focussing on resource use and outcomes at baseline, 1, 6 and 12 months. Questionnaires will be administered in person, by telephone or otherwise remotely (using MS Teams and REDCap software) to maximise response rates. For consistency and comparability, follow up questionnaires will adopt the same mode of administration as baseline, as far possible. Eligibility criteria for the questionnaire study are shown in Table 1.

Table 1: Eligibility Criteria for questionnaire study

| Inclusion criteria | Exclusion criteria |
|---|---|
| Aged over 18 years | Ongoing participation in multiple CLOs |
| Community participant associated with participating CLOs | One-time participation only |
| Participant in an activity that involves several contacts over time (CLOs might provide one-time advice services for example but, for comparability, we will | Not within the community of the CLO. |

For peer review only - http://bmjopen.bmj.com/site/about/guidelines.xhtml

| | |
|---|---|
| focus on a period of consistent participation). | |

Eligible participants who express interest in taking part in the study via the CLO staff will be contacted by researchers to provide study information, complete consent procedures and administer a baseline questionnaire comprised of sociodemographic questions, resource use questions and a number of standardised outcome measures.

The primary outcome measure for the questionnaire study is the ICECAP-A (ICEpop CAPability measure for adults[32]. The ICECAP-A has been selected for three important reasons. Firstly, the dimensions of the ICECAP-A map well onto the intermediate outcomes identified in previous empirical research and in practice-based models synthesised in our conceptual model (see Figure 3) and so we set out to measure things that have been shown to be important to beneficiaries, practitioners and according to theory. Secondly it is validated and is supported for use in economic evaluation by NICE[33] and thirdly preference-based population tariffs are available[34]. Our secondary outcome measures are health status using EQ-5D 5L[35], Warwick Edinburgh Mental Wellbeing Scale (WEMWBS)[36] , and Social Connectedness [37].

There is no estimate of minimal important difference available from existing datasets of ICECAP-A, so the study will be designed to detect a commonly used standardised effect size of 0.25 at 6 months. This represents a small to medium effect size. A sample size of 252 will be required to provide 80% power at 5% significance (with two-sided alpha) to detect a difference of 0.25 SDs. We will recruit 360 to achieve an effective sample of 252 completed questionnaires at 6 months, which allows for credible retention of 70%. We anticipate 60 to 65% retention at 12 months (n=225).

A statistical analysis plan will be agreed before database lock. Descriptive statistics will be used to explore and analyse the data for associations. Random effects regressions with clustered standard errors will be employed to account for the longitudinal nature of our data and for the fact individuals are clustered in CLOs. Multilevel modelling that explicitly acknowledges the hierarchical clustering of the data will be employed[38,39]. These models will estimate the impact of the CLOs on the outcome measures over time after controlling for individual and CLO characteristics. The analyses will be run for our primary and secondary outcomes separately.

### *Q Methodology study*

The aim of all Q studies is to identify patterns of shared perspectives[40–42]. In the context of realist methods this sits well with the goal of uncovering regularities in data[43]. We will make use of this approach to unpack the mechanisms at work in different contexts[44]. The Q set of statements will comprise of *candidate mechanisms* drawn from theory, interview and workshop data and hypotheses that emerge from preliminary analysis of context-outcome patterns in the questionnaire study.

Up to 60 participants, selected from the questionnaire sample to cover different contexts and outcomes, will complete Q sorts to identify which mechanisms they consider help to explain

For peer review only - http://bmjopen.bmj.com/site/about/guidelines.xhtml

why outcomes arise (or do not) in context. Different mechanisms might relate to the same outcomes in different contexts and we will gather descriptive information about Q participants' contexts. Factor analysis of Q sort data based on correlations between individuals' Q sorts will reveal shared patterns of context and mechanisms for interpretation.

### *Economic analysis*

The economic evaluation will take the form of a cost consequence analysis (CCA). A CCA presents costs and outcomes in a disaggregated form which is appropriate given the multi-sectoral context in which the CLOs operate and the range of outcomes of importance[45]. We will seek to quantify resource implications arising from both contextual (CM) and programme mechanisms (PM), and how these relate to agents' responses (A) and outcomes (O) measured.

The first stage of the economic analysis will be to map out the resource inputs required to deliver the activities provided by the CLOs. This will be done in collaboration with the CLOs and will be assessed and updated throughout the project. All resource inputs will be totalled and turned into a cost per participant. Secondly, as part of the questionnaire study, participants will self-report use of other health care, social and community-based services in the month prior to joining the CLO and then at 1, 6 and 12-month follow up. This will allow us to assess changes in resource use within the study participant group in the form of a before-and-after design. Units of each item will be recorded and presented along with unit costs deriving from local publicly available sources[46].

The resource use will be presented alongside the estimates of health and wellbeing outcomes from the primary and secondary outcome measures and with qualitative data from the interviews conducted in both phase 1 and 2 of the study in the CCA.

### *Sustainability and scalability*

Building on information deriving from our economic analysis and relevant financial documents (e.g. contracts and agreements) provided by CLOs managers, our assessment of financial sustainability and scalability of CLOs will be informed by interviews with CLO stakeholders (e.g. managers, funders). Combining economic evaluation with qualitative interview data will help to better understand *processes* associated with developing sustainable CLOs. The interviewees will be asked about best practices and key challenges involved in establishing, operating and growing sustainable CLOs, and what it means to be 'sustainable' in their specific community contexts. Previous research[47] suggests that sustainability and scalability in a community context is rarely straightforward and CLOs frequently face difficulties in maintaining and sustaining their services and activities.

Drawing on a sample of diverse stakeholders from a range of CLOs (i.e. variation across rural/urban, organisational size and maturity, range of activities etc.), will help us to explore how different contexts can promote or inhibit particular mechanisms; intended and unintended outputs and outcomes; income streams and views about sustainability.

For peer review only

## Patient and Public Involvement

Public involvement in the preparation and design of the project as presented to funders was achieved through co-investigators and collaborators in the community sector who work in and with CLOs and are co-authors on this paper.

Patient and public involvement and engagement (PPIE) has been integrated throughout the project by establishing a panel of individuals with experience of living in our CLO communities. The 'Lived Experience Panel' (LE Panel) will ensure that the project is informed and guided by ongoing community expertise, perspective and voice and that findings are relevant and meaningful to community organisations.

The LE Panel will comprise representatives of the CLOs, identified and recruited with the support of our community partners. The LE Panel will meet at key stages of the research process to guide and inform the research questions, methods, data analysis and interpretation of results – with space for ongoing involvement and communication between Panel meetings. The hands-on nature of these participatory methods and the support of the research team will allow participants to actively engage with the research, placing more value on their expertise. Representatives of the LE Panel will also be members of the Study Steering Committee, complementing the perspectives of other key stakeholders.

Training opportunities will be provided allowing participants to build their skills and knowledge around community-based research, promoting two-way learning exchange between the researchers and Panel participants, which will elevate community voices and strengthen researcher-participant relations. Continuous evaluation will be fostered to improve facilitation and promote learning throughout the life and work of the Panel

## Ethics and dissemination

This project has been approved by the Glasgow Caledonian University School of Health and Life Sciences Ethics Committee on 15/7/21 (ref HLS/NCH/20/034) and subsequently affirmed by Ethics Committees at Bournemouth University, Queens University Belfast (MHLS 21_94) and University of East London (ETH2021-0226). Common Health Assets does not involve any NHS sites, staff or patients. IRAS and NHS REC are not required. The project is funded for three years beginning 1st September 2021.

This project will advance the research field, at the nexus of public health research and community development/social enterprise research and methodologically in terms of economics and quantitative approaches in realist evaluation and in the use of Q methodology to explore mechanisms. Our PPIE strategy including lived experience panel, stakeholder involvement in the research team and Study Steering Committee (SSC), and policy briefing will ensure that the research involves users of evidence throughout to enhance the relevance and application to policy and practice. CLOs will be able to use research findings as part of their impact evidence base when applying for public funding or in refining their activities where appropriate. We will disseminate our findings using a range of media, and pitched to suit the needs of our three main constituencies of interest; namely: (i) Practitioners and activists; (ii) Policymakers and Commissioners; and (iii) Academics.

For peer review only

The project outputs will comprise peer reviewed papers in academic journals, blogs, short videos, photography exhibition, policy briefing papers, website (www.commonhealthassets.uk) and a commissioning guide. We will hold workshops to share findings and develop recommendations with practitioners and policy actors. Each CLO will receive a report summarising the study findings and their own local findings where possible.

## Authors' contributions

This paper is based on a longer protocol approved by the NIHR and drafted by all co-investigators. Rachel Baker led on the design of the research and the writing of the protocol, producing the initial draft. MB, JC, CD, JF, KG, JMc, SM, HM, AM, AP, SP, MR, PS, MS, AS and MK contributed to the NIHR grant application, the NIHR approved protocol and revised drafts and agreed final text of this paper. MA, RC, EC, AE, LAF, AG, AMc, JM, LOH, and JR contributed to the NIHR approved protocol, revised drafts and agreed final text of this paper.

## Funding

This study is funded by the National Institute for Health Research (NIHR) Public Health Research programme (NIHR 129118]. The views expressed are those of the author(s) and not necessarily those of the NIHR or the Department of Health and Social Care.

## Conflicts of interests statement

None of the authors have a financial interest in the results of this research.

## Data Statement

We intend to publish our data to a recognised UK data repository if it is practically possible to do. Data sharing statements will be included when publishing the findings of this research which will describe how to access the data. The sharing of data will be done in the most appropriate way and only if it is clear that sharing will help deliver research that maximises benefits to the public. Final decisions with respect to data sharing will be taken with the following in mind: protect the confidentiality and privacy of individuals; respect the terms of consent by individuals who are involved in the study; be consistent with relevant legal, ethical and regulatory frameworks; and guard against unreasonable resource use to share the data. Researchers and other users will have to apply to gain access to the data and the process of gaining access will be governed by Glasgow Caledonian University and NIHR data sharing guidelines.

## Acknowledgements

The proposed research would not be possible without the partnership of our community led organisations and their community participants and volunteers, without whom the research would be impossible. Their commitment and collaboration are particularly appreciated given the Covid-19 pandemic, during which we planned to start this work, when CLOs were working hard to support their communities.

For peer review only

Figure 1 – Study illustration: phase 1 and phase 2 methods and outputs
Figure 2-'Plumbing Diagram' showing how data sources feed into the realist framework connecting context, mechanisms and outcomes
Figure 3 Conceptual Model - a synthesis of research and practice-based models

For peer review only

# References

[1] E. Blas, L. Gilson, M.P. Kelly, R. Labonté, J. Lapitan, C. Muntaner, P. Östlin, J. Popay, R. Sadana, G. Sen, Addressing Social Determinants of Health Inequities: What Can the State and Civil Society Do?, The Lancet. 372 (2008) 1684–1689.

[2] F. Garven, L. Pattoni, J. McLean, Asset-Based Approaches: Their Rise, Role and Reality, Dunedin Academic Press, Edinburgh, London, 2016.

[3] M.J. Roy, The Assets-Based Approach: Furthering a Neoliberal Agenda or Rediscovering the Old Public Health? A Critical Examination of Practitioner Discourses, Crit. Public Health. 27 (2017) 455–464.

[4] NHS England, Five Year Forward View, NHS England, London, 2014. https://www.england.nhs.uk/wp-content/uploads/2014/10/5yfv-web.pdf.

[5] VCSE Sector, Department of Health, NHS England, Public Health England, The Joint VCSE Review Action Plan, Department of Health, London, 2018. https://voluntarycommunitysocialenterprisereview.files.wordpress.com/2019/03/joint-vcse-review-action-plan-2018.pdf (accessed April 4, 2019).

[6] Public Health England, A Guide to Community-Centred Approaches for Health and Wellbeing, Public health England, London, 2015. https://assets.publishing.service.gov.uk/government/uploads/system/uploads/attachment_data/file/768979/A_guide_to_community-centred_approaches_for_health_and_wellbeing__full_report_.pdf (accessed July 9, 2019).

[7] Renewing Scotland's public services: priorities for reform in response to the Christie Commission, (n.d.). http://www.gov.scot/publications/renewing-scotlands-public-services-priorities-reform-response-christie-commission/ (accessed September 28, 2022).

[8] T. Van Bortel, N.D. Wickramasinghe, A. Morgan, S. Martin, Health assets in a global context: a systematic review of the literature, BMJ Open. 9 (2019) e023810.

[9] J. McLean, V. McNeice, C. Mitchell, Asset-Based Approaches in Service Setting: Striking a Balance, Glasgow Centre for Population Health, Glasgow, 2017. https://www.gcph.co.uk/assets/0000/6049/Asset_based_approaches_in_service_settings.pdf (accessed July 24, 2019).

[10] Glasgow Centre for Population Health, Scottish Community Development Centre, Positive Conversations, meaningful change: learning from Animating Assets, 2015. https://www.gcph.co.uk/assets/0000/5433/0356_learning_from_animating_assets_web.pdf (accessed September 22, 2022).

[11] B. Fisher, Community Development in Health: A Literature Review, 2016.

[12] Tailor-made: how community groups improve people's lives, Trust Lond. (n.d.). https://www.trustforlondon.org.uk/publications/tailor-made-how-community-groups-improve-peoples-lives/ (accessed September 22, 2022).

[13] K. Skivington, L. Matthews, S.A. Simpson, P. Craig, J. Baird, J.M. Blazeby, K.A. Boyd, N. Craig, D.P. French, E. McIntosh, M. Petticrew, J. Rycroft-Malone, M. White, L. Moore, A new framework for developing and evaluating complex interventions: update of Medical Research Council guidance, BMJ. 374 (2021) n2061.

For peer review only

[14] D. Kelly, A. Steiner, M. Mazzei, R. Baker, Filling a void? The role of social enterprise in addressing social isolation and loneliness in rural communities, J. Rural Stud. 70 (2019) 225–236.

[15] F. Calo, M.J. Roy, C. Donaldson, S. Teasdale, S. Baglioni, Exploring the contribution of social enterprise to health and social care: A realist evaluation, Soc Sci Med. 222 (2019) 154–161. https://doi.org/10.1016/j.socscimed.2019.01.007.

[16] B. Macaulay, M. Mazzei, M.J. Roy, S. Teasdale, C. Donaldson, Differentiating the effect of social enterprise activities on health, Soc. Sci. Med. 200 (2018) 211–217.

[17] M.J. Roy, R. Baker, S. Kerr, Conceptualising the Public Health Role of Actors Operating Outside of Formal Health Systems: The Case of Social Enterprise, Soc. Sci. Med. 172 (2017) 144–152. https://doi.org/10.1016/j.socscimed.2016.11.009.

[18] R. Pawson, N. Tilley, Realistic Evaluation, Sage, Thousand Oaks, CA, 1997.

[19] S. Porter, Realist Evaluation: An Immanent Critique, Nurs. Philos. 16 (2015) 239–251.

[20] English indices of deprivation 2019: mapping resources, GOV.UK. (n.d.). https://www.gov.uk/guidance/english-indices-of-deprivation-2019-mapping-resources (accessed September 28, 2022).

[21] Scottish Index of Multiple Deprivation 2020, (n.d.). https://www.gov.scot/collections/scottish-index-of-multiple-deprivation-2020/ (accessed September 28, 2022).

[22] Northern Ireland Multiple Deprivation Measure 2017 (NIMDM2017), North. Irel. Stat. Res. Agency. (n.d.). https://www.nisra.gov.uk/statistics/deprivation/northern-ireland-multiple-deprivation-measure-2017-nimdm2017 (accessed September 28, 2022).

[23] B. Macaulay, M.J. Roy, C. Donaldson, S. Teasdale, A. Kay, Conceptualizing the Health and Well-Being Impacts of Social Enterprise: A UK-Based Study, Health Promot. Int. 33 (2018) 748–759.

[24] A. Morgan, M. Davies, E. Ziglio, eds., Health Assets in a Global Context: Theory, Methods, Action: Investing in Assets of Individuals, Communities and Organizations, Springer, New York, 2010.

[25] A. Morgan, E. Ziglio, Revitalising the Evidence Base for Public Health: An Assets Model, Glob. Health Promot. 14 (2007) 17–22.

[26] F. Calò, M.J. Roy, C. Donaldson, S. Teasdale, S. Baglioni, Exploring the Contribution of Social Enterprise to Health and Social Care: Approaches and Considerations, Soc. Enterp. J. 17 (2021) 140–155.

[27] L.A. Munford, M. Sidaway, A. Blakemore, M. Sutton, P. Bower, Associations of Participation in Community Assets with Health-Related Quality of Life and Healthcare Usage: A Cross-Sectional Study of Older People in the Community, BMJ Open. 7 (2017) e012374.

[28] E. Bellazzecca, R. Baker, M. Bertotti, K. Galway, A. Morgan, L. O'Hare, M.J. Roy, S. Porter, CommonHealth Assets: a protocol for a realist review of reviews of the health and wellbeing impact of community led organisations in disadvantaged communities. PROSPERO 2021 CRD42021287959, (2021). https://www.crd.york.ac.uk/prospero/display_record.php?RecordID=287959 (accessed September 28, 2022).

[29] B.M.A. Wang Caroline, Photovoice: Concept, Methodology, and Use for Participatory Needs Assessment, Health Educ. Behav. 24 (1997) 369–387.

[30] QSR International Pty Ltd, NVivo, (2020). https://www.qsrinternational.com/nvivo-qualitative-data-analysis-software/home?_ga=2.78631316.1092956994.1664355022-573106870.1664355022.

[31] M.R. Roller, P.J. Lavrakas, Applied qualitative research design: A total quality framework approach, The Guilford Press, New York, NY, US, 2015.

[32] H. Al-Janabi, T. N Flynn, J. Coast, Development of a self-report measure of capability wellbeing for adults: the ICECAP-A, Qual. Life Res. 21 (2012) 167–176.

[33] 7 Incorporating economic evaluation | The social care guidance manual | Guidance | NICE, (n.d.). https://www.nice.org.uk/process/pmg10/chapter/incorporating-economic-evaluation (accessed September 28, 2022).

[34] T.N. Flynn, E. Huynh, T.J. Peters, H. Al-Janabi, S. Clemens, A. Moody, J. Coast, Scoring the Icecap-a capability instrument. Estimation of a UK general population tariff, Health Econ. 24 (2015) 258–269.

[35] EuroQol Group, EuroQol--a new facility for the measurement of health-related quality of life, Health Policy Amst. Neth. 16 (1990) 199–208.

[36] R. Tennant, L. Hiller, R. Fishwick, S. Platt, S. Joseph, S. Weich, J. Parkinson, J. Secker, S. Stewart-Brown, The Warwick-Edinburgh Mental Well-being Scale (WEMWBS): Development and UK validation, Health Qual. Life Outcomes. 5 (2007) No Pagination Specified-No Pagination Specified.

[37] R.M. Lee, S.B. Robbins, Measuring belongingness: The Social Connectedness and the Social Assurance scales, J. Couns. Psychol. 42 (1995) 232–241.

[38] H. Goldstein, Multilevel Statistical Models, John Wiley & Sons, 2011.

[39] F. Steele, Multilevel models for longitudinal data, J. R. Stat. Soc. Ser. A Stat. Soc. 171 (2008) 5–19.

[40] R. Baker, C. Thompson, R. Mannion, Q methodology in health economics, J. Health Serv. Res. Policy. 11 (2006) 38–45.

[41] S. Watts, P. Stenner, Doing Q Methodological Research; Theory method and interpretation, SAGE Publications Ltd, London, 2012.

[42] J. Van Exel, G. De Graaf, Q methodology: A sneak preview, Retrieved January. 24 (2005). https://www.researchgate.net/profile/Gjalt_Graaf/publication/228574836_Q_Methodology_A_Sneak_Preview/links/02bfe50f946fc9978b000000.pdf (accessed August 31, 2017).

[43] J. Duncan Millar, H. Mason, L. Kidd, IMPETUS Research Team, What is important in supporting self-management in community stroke rehabilitation? A Q methodology study, Disabil. Rehabil. (2022) 1–9.

[44] B. Astbury, Making Claims Using Realist Methods, in: N. Emmel, J. Greenhalgh, A. Manzano, M. Monaghan, S. Dalkin (Eds.), Doing Realist Res., SAGE Publications Ltd, Los Angeles, 2018: pp. 59–78.

[45] R.T. Edwards, E. McIntosh, eds., Applied Health Economics for Public Health Practice and Research, Oxford University Press, Oxford, New York, 2019.

[46] C.L. Curtis, A. Burns, Unit Costs of Health and Social Care 2018, (2018) 203.

[47] F. Lyon, H. Fernandez, Strategies for scaling up social enterprise: lessons from early years providers, Soc. Enterp. J. 8 (2012) 63–77.

For peer review only

**Figure 1 – Study illustration: phase 1 and phase 2 methods and outputs**

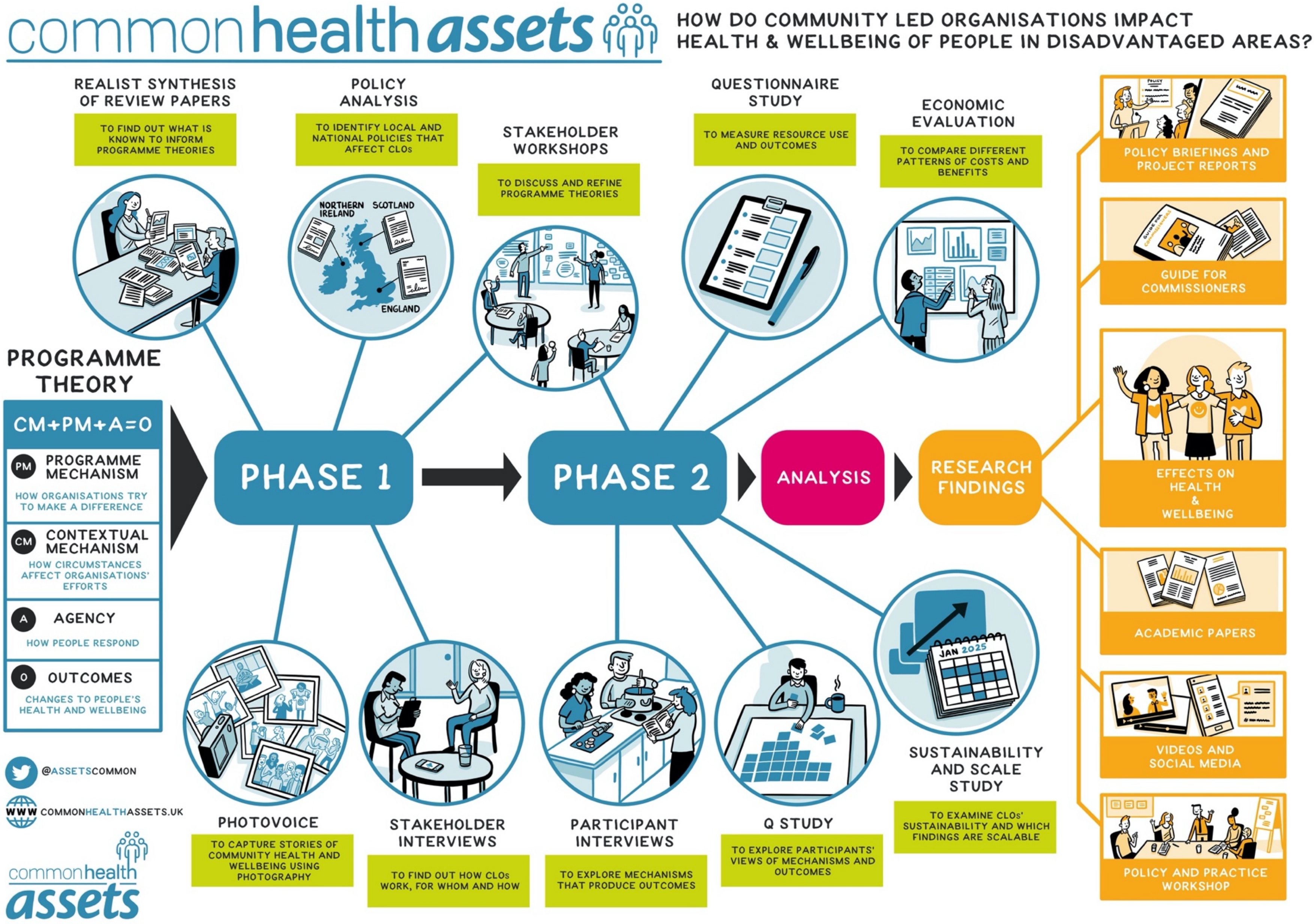

FIGURE 2–'PLUMBING DIAGRAM' SHOWING HOW DATA SOURCES FEED INTO THE REALIST FRAMEWORK CONNECTING CONTEXT, MECHANISMS AND OUTCOMES.

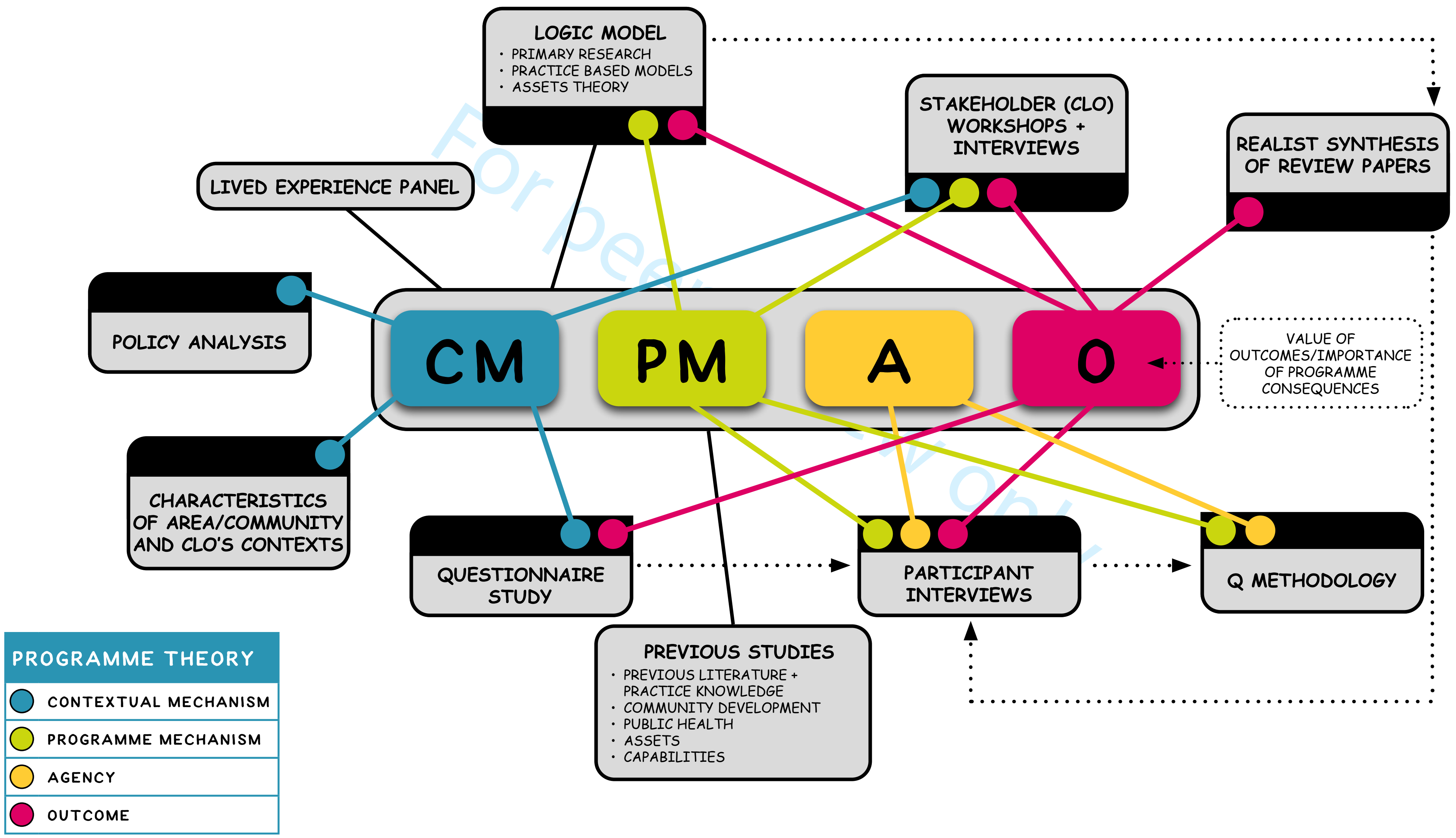


For peer review only - http://bmjopen.bmj.com/site/about/guidelines.xhtml

**REFERENCE:** PORTER, S. (2015) REALIST EVALUATION: AN IMMANENT CRITIQUE, NURSING PHILOSOPHY, 16, 239–251

# FIGURE 3 CONCEPTUAL MODEL – A SYNTHESIS OF RESEARCH AND PRACTICE-BASED MODELS.

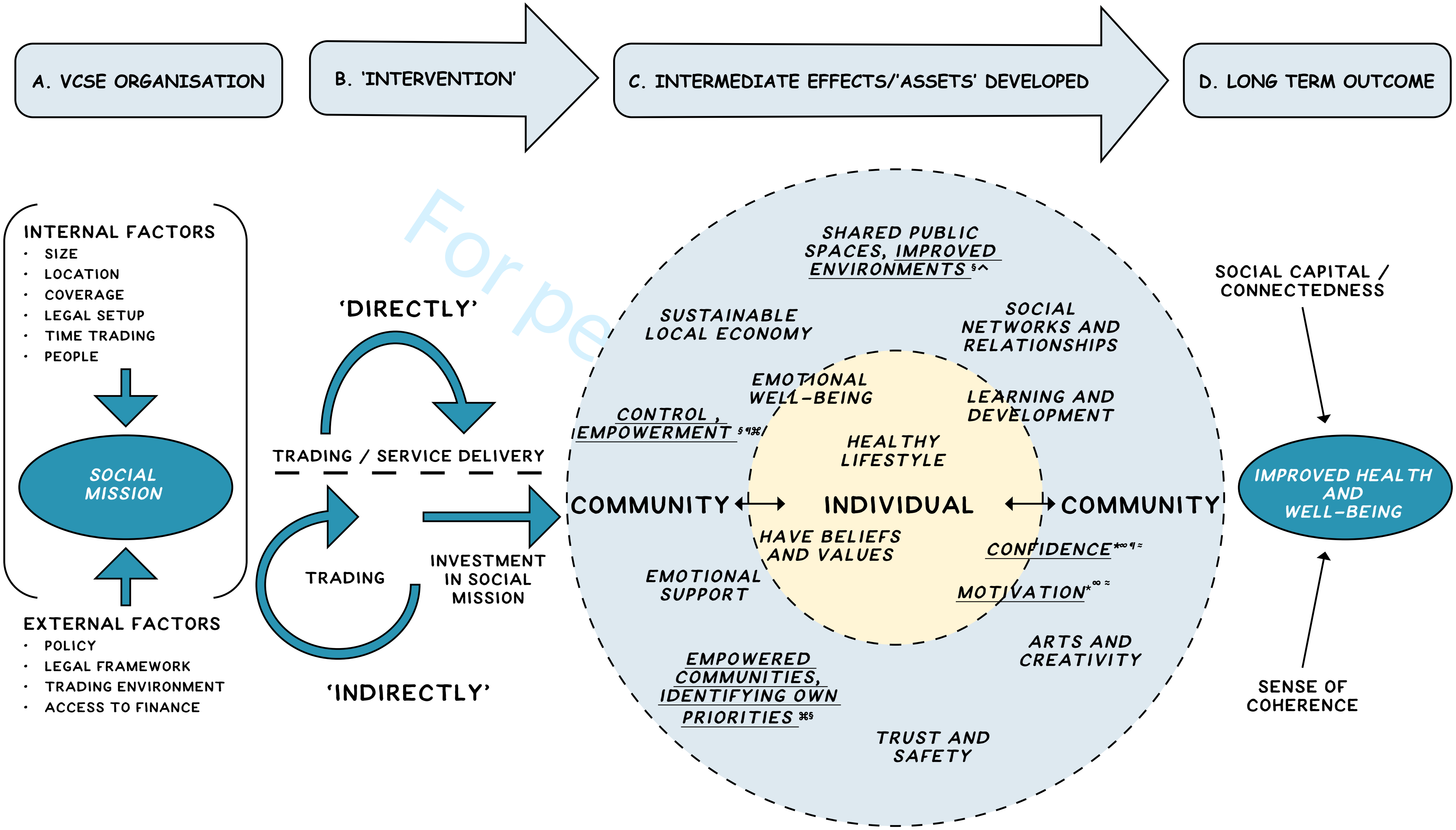


**KEY TO MODEL WITH ADDITIONS TO COMMONHEALTH CONCEPT MODEL, FOLLOWING SYNTHESIS UNDERLINED:**

CALO ET AL (2018); ∞KELLY ET AL (2019); §MACAULEY ET AL (2017); ≈ROY ET AL (2017); ^HEALTH ASSETS MODEL (MORGAN ET AL 2007, 2010)

¶NNHSA MODEL OF HEALTH CREATION; ⌘SCDC COMMUNITY LED HEALTH MODEL.
