# Supplementary material for: Common health assets protocol: a mixed-methods, realist evaluation and economic appraisal of how community-led organisations (CLOs) impact on the health and well-being of people living in deprived areas
Source: BMJ Open. 2023 Mar 15;13(3):e069979. doi: 10.1136/bmjopen-2022-069979 (PMC10030928; doi:10.1136/bmjopen-2022-069979)
Supplement: Reviewer comments [file bmjopen-2022-069979.reviewer_comments.pdf]

PEER REVIEW HISTORY

BMJ Open publishes all reviews undertaken for accepted manuscripts. Reviewers are asked to complete a checklist review form (**http://bmjopen.bmj.com/site/about/resources/checklist.pdf**) and are provided with free text boxes to elaborate on their assessment. These free text comments are reproduced below.

ARTICLE DETAILS

**TITLE (PROVISIONAL)**	Common Health Assets protocol: a mixed-methods, realist evaluation and economic appraisal of how community-led organisations (CLOs) impact on the health and wellbeing of people living in deprived areas
**AUTHORS**	Baker, Rachel; Ahmed, Mohasin; Bertotti, Marcello; Cassidy, John; Chipuriro, Rejoice; Clewett, Emma; Donaldson, Cam; Elders, A; Fenge, Lee Ann; Fox, Julie; Galway, K.; Gildea, Aideen; McGuiness, Aileen; McLean, Jennifer; Manoukian, Sarkis; Mason, Helen; Morgan, A; Mulholland, Jill; O'Hare, Liam; Paterson, Andrew; Porter, Sam; Rendall, Jack; Roy, Michael; Seaman, Peter; Simpson, Merron; Steiner, Artur; Kelly, Michael

This article was not externally reviewed at BMJ Open. Protocols that have been independently assessed prior to submission to BMJ Open are usually fast-tracked to publication on the grounds that further substantial changes will not be possible. This independent assessment will usually be external, independent review for both a substantial grant award from a non-commercial or government funder and ethics approval.
